# Differential Blood and Mucosal Immune Responses against an HIV-1 Vaccine Administered via Inguinal or Deltoid Injection

**DOI:** 10.1371/journal.pone.0088621

**Published:** 2014-02-18

**Authors:** Otto O. Yang, F. Javier Ibarrondo, Charles Price, Lance E. Hultin, Julie Elliott, Patricia M. Hultin, Roger Shih, Mary Ann Hausner, Hwee L. Ng, Jennifer Hoffman, Beth D. Jamieson, Peter A. Anton

**Affiliations:** 1 Department of Medicine and University of California Los Angeles AIDS Institute, David Geffen School of Medicine at University of California Los Angeles, Los Angeles, California, United States of America; 2 Department of Microbiology, Immunology, and Molecular Genetics, David Geffen School of Medicine at University of California Los Angeles, Los Angeles, California, United States of America; 3 AIDS Healthcare Foundation, Los Angeles, California, United States of America; Rush University, United States of America

## Abstract

Mucosal immunity is central to sexual transmission and overall pathogenesis of HIV-1 infection, but the ability of vaccines to induce immune responses in mucosal tissue compartments is poorly defined. Because macaque vaccine studies suggest that inguinal (versus limb) vaccination may better target sexually-exposed mucosa, we performed a randomized, double-blinded, placebo-controlled Phase I trial in HIV-1-uninfected volunteers, using the recombinant Canarypox (CP) vaccine vCP205 delivered by different routes. 12 persons received vaccine and 6 received placebo, divided evenly between deltoid-intramuscular (deltoid-IM) or inguinal-subcutaneous (inguinal-SC) injection routes. The most significant safety events were injection site reactions (Grade 3) in one inguinal vaccinee. CP-specific antibodies were detected in the blood of all 12 vaccinees by Day 24, while HIV-1-specific antibodies were observed in the blood and gut mucosa of 1/9 and 4/9 evaluated vaccinees respectively, with gut antibodies appearing earlier in inguinal vaccinees (24–180 versus 180–365 days). HIV-1-specific CD8^+^ T lymphocytes (CTLs) were observed in 7/12 vaccinees, and blood and gut targeting were distinct. Within blood, both deltoid and inguinal responders had detectable CTL responses by 17–24 days; inguinal responders had early responses (within 10 days) while deltoid responders had later responses (24–180 days) in gut mucosa. Our results demonstrate relative safety of inguinal vaccination and qualitative or quantitative compartmentalization of immune responses between blood and gut mucosa, and highlight the importance of not only evaluating early blood responses to HIV-1 vaccines but also mucosal responses over time.

**Trial Registration:**

ClinicalTrials.gov NCT00076817

## Introduction

As of 2010, 34 million people were living with HIV-1 infection and 2.7 million new infections occurred that year alone (UNAIDS World AIDS Day report 2011). Although antiretroviral therapy (ART) is effective, it is costly, and requires lifelong administration and continuous monitoring, which is limiting in resource-poor endemic regions. Thus, the development of a safe and effective vaccine against HIV-1 remains a critical goal to stem the pandemic. Of over 30 vaccine candidates tested in human trials, only one has shown a hint of efficacy [1] in preventing HIV-1 acquisition, and none have had any effect on immune control after infection [2].

The vast majority of HIV-1 transmissions occur through sexual contact and exposure of mucosal surfaces. Mucosal tissues of the genital and intestinal tracts are pro-inflammatory environments rich in activated CD4^+^ T-cells, which are the preferred targets for HIV-1 infection. Numerous studies in non-human primates and humans have demonstrated that the gut mucosa, which contains about the 50% of total body lymphocytes [3], is the predominant site of early HIV-1 replication and amplification regardless the route of infection[4]. Moreover, the mucosal immune system is compartmentalized; immune responses to the same antigen(s) can differ between anatomic compartments in terms of specificity, avidity and memory T cell phenotypes [5]–[7]. Thus it is clear that the mucosa is a key site for eliciting protective immunity by novel vaccine strategies against HIV-1.

Systemic immunization has been proven to be adequate for most vaccines, including some against mucosal pathogens. There is evidence, however, that mucosal immunity can play an important role in protection but is dependent on the route of vaccine administration. Oral polio vaccine (live attenuated) generates gut mucosal immunity that limits subsequent shedding of poliovirus after infection, while shedding in stool is noted after vaccination via deltoid intramuscular injection (inactivated), although both vaccines prevent systemic dissemination and poliomyelitis [8]. Murine and macaque vaccination models indicate compartmentalization of the immune system and the potential importance of the route of vaccine delivery [5], [9], [10]. Here, we utilize the HIV-1-recombinant Canarypox vaccine ALVAC-HIV vCP205 to examine blood versus gut mucosal immune responses when the vaccine is delivered via two different vaccination routes: deltoid/intramuscular (deltoid-IM) versus inguinal/subcutaneous (inguinal-SC).

## Materials and Methods

The protocol for this trial and supporting CONSORT checklist are available as supporting information; see Checklist S1 and Protocol S1.

### Ethics Statement

This study was approved by the UCLA Office of the Human Research Protection Program Institutional Review Board (UCLA IRB #10-000520) with all participants providing written informed consent.

### Objectives

The objectives of this Phase 1 trial were to (i) evaluate the safety of inguinal immunization using an already human-evaluated HIV-1 vaccine [11], [12], (ii) define and compare differences in immune responses to the vaccine carrier (canarypox) and HIV-1 proteins in blood and gastrointestinal mucosal biopsy samples. The working hypotheses were that the inguinal immunization route would be safe, that both mucosal antibody and CD8^+^ T lmphocyte responses would be detectable in gut mucosa and blood, and that blood and gut mucosa responses would differ. The protocol was designed by the investigators with collaborative input and IND-support from Aventis Pasteur (now Sanofi Pasteur). This Phase 1 interventional clinical trial started recruitment in October 2003, enrolling the first subject 11/17/03 and ending follow-up of the last patient 7/27/05. This predated the requirements for pre-registration with ClinicalTrials.gov (7/1/05) and CONSORT (www.consort-statement.org) compliance. However, this study was registered with ClinicalTrials.gov on 3/4/04 (NCT00076817).

### Study subjects

Study inclusion criteria included willingness to avoid any rectal insertions one week prior to vaccination and one week before/after each flexible sigmoidoscopy. Exclusion criteria included HIV-1 infection, any chronic gastrointestinal disorder, history of significant gastrointestinal bleeding, or other significant medical disorders. Enrollment was protocol-defined as having met initial screening criteria, providing written informed consent, and having negative evaluations for HIV-1 or sexually transmitted infections (syphilis, *Chlamydia trachomatis* and *Neisseria gonorrhea* and active *Herpes simplex* lesions). Female participants were required to be using an acceptable form of contraception. Prospective vaccinees were briefed on the risks and benefits of the ALVAC vCP205 candidate vaccine (Sanofi Pasteur) and the potential implications vaccine-induced positive HIV-1 serology [13]. 22 men and women aged 25–60 years were enrolled; 18 persons met randomization criteria and proceeded to receive vaccinations.

### Vaccine

The live recombinant canarypox vaccine ALVAC vCP205 containing HIV-1 IIIB *env/gag/protease* was produced under GMP conditions and provided by Sanofi Pasteur. The IND application to the FDA for a new site of administration (inguinal-subcutaneous) was supported by Sanofi Pasteur and held by Dr. Anton/UCLA. AP also provided placebo vaccine, a mixture of virus stabilizer and freeze-drying medium with a diluent for reconstitution. The diluent was 1 mL of sterile pyrogen-free 0.4% sodium chloride.

### Study design

**Figure 1:**
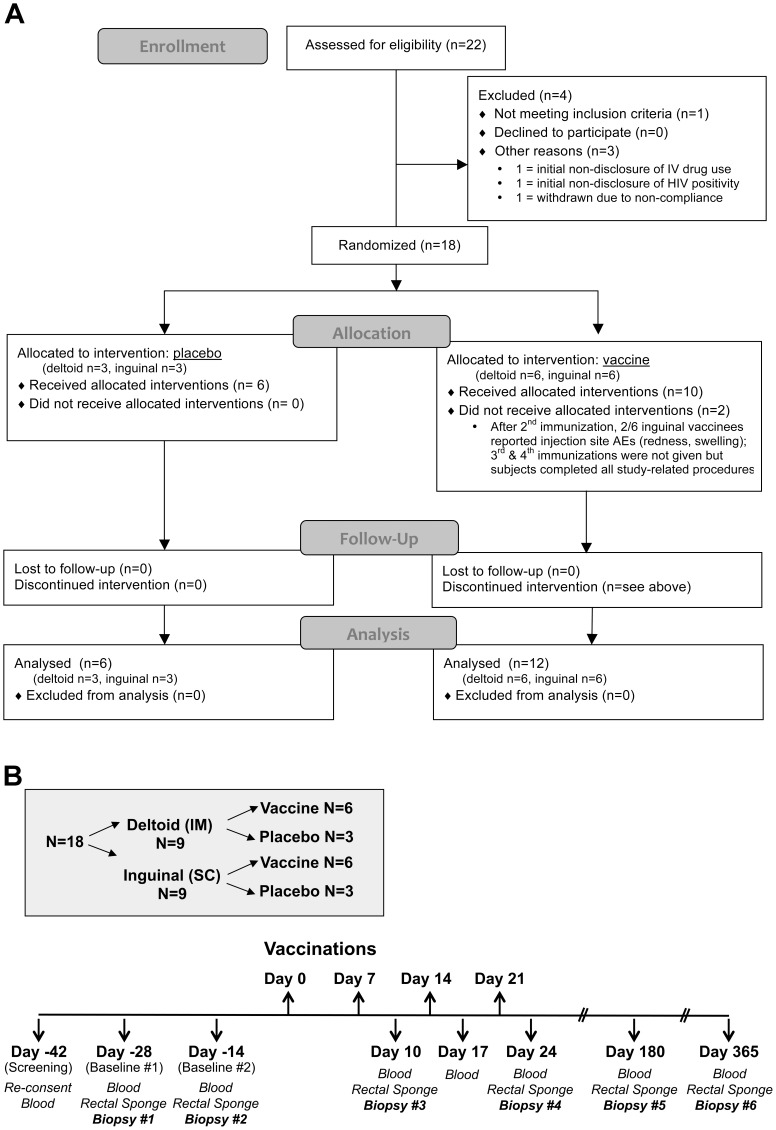
Clinical trial design. A. CONSORT flowchart of subject enrolment. B. After an initial screening visit, blood and gut mucosal biopsies were obtained 28 and 14 days before initiation of vaccination. Vaccinations (vCP205 or placebo) were administered Days 0, 7, 14, 21. Blood samples then were collected on Days 10, 17, 24, 180, and 360. Gut mucosal biopsies were collected at Days 10, 24, 180, 360.

This was a single site, double-blinded, placebo-controlled, randomized (2∶1; vaccine: placebo), Phase 1 trial of the vCP205 vaccine administered via deltoid intramuscular (deltoid-IM) versus inguinal subcutaneous (inguinal-SC) vaccinations. Participants were defined as “enrolled” after completing baseline examinations but prior to receiving the first vaccination. Randomization, which was not stratified by any baseline covariate, was performed by a study statistician working directly with the research pharmacy. Participants were randomized first to receive either placebo (n = 6) or vCP205 vaccine (n = 12). The subjects within each of those groups then were randomized into equal numbers to receive injections either via deltoid-intramuscular or inguinal-subcutaneous routes (placebo deltoid n = 3, placebo inguinal n = 3, vaccine deltoid n = 6, vaccine inguinal n = 6). All vaccinations were administered in a double-blinded fashion (the syringes and their contents were visually indistinguishable between placebo and vaccine), and all study staff remained blinded to randomization codes until data lockdown by the study statistician following the pre-determined data quality management protocol. Plasma HIV-1 RNA was measured at each study visit to detect any interval/intercurrent infections. Participants were given a symptom diary and encouraged to call/report any unexpected symptoms, and were called daily by the study coordinator for the week following each vaccination. The primary objective was to determine the safety profile of the vaccine. Secondary objectives were to determine: (i) whether deltoid and inguinal vaccinations induced differential immune responses (humoral and cellular); (ii) if detectable mucosal responses arose; and (iii) whether mucosal responses varied by vaccination route and matched those seen in blood. The overall study design is summarized in Figure 1.

### Vaccination schedule

Following two baseline mucosal and blood sample acquisitions (at Day -28 and Day -14, prior to first immunization), vaccinations were administered at week 0 (10^6^ TCID_50_) and then weekly for 3 weeks (10^7^ TCID_50_). Inguinal-SC immunizations were administered by injection medial to the femoral vein, a modification of a previously described, targeted iliac lymph node (TILN) protocol [14]. Deltoid-IM immunizations were delivered per routine clinical protocols. Both deltoid-IM and inguinal-SC vaccinations were alternatively administered to the left and right limbs.

### Mucosal sampling

Mucosal sampling was performed as previously described [15]–[17] during the two baseline visits and then three days (±2 day window) after the subsequent 3 vaccinations, and finally at Day 180 and Day 365 after the first vaccination. During each sampling, anoscopy was first performed for placement of two, pre-moistened surgical sponges (Ultracell ® Medical Technologies, North Stonington, CT) for 5 minutes to collect mucosal secretions for antibody quantification [18]. Flexible sigmoidoscopy was then performed with 20 biopsies acquired at approximately 30 cm from the anal verge as previously described [15], [17], [19], for isolation of mucosal mononuclear cells (MMC). Briefly, biopsies (8 mm×2 mm×1 mm from large-cup, endoscopic biopsy forceps (Microvasive Radial Jaw #1589, outside diameter 3.3 mm) were taken and immediately placed into 15 ml of tissue culture medium (RPMI 1640, Irvine Scientific).

### Elution of rectal secretions from surgical sponges

Elution of rectal secretions from the surgical sponges was performed with minor modifications of a previously described protocol [18]. Briefly, collected sponges were immediately transported to the laboratory on ice and frozen at −80°C for later batch processing. Sponge contents were eluted twice with 250 µl cold PBS containing 0.25% BSA (Sigma Chemicals, St Louis, MO), 1% Igepal (Sigma Chemicals, St. Louis, MO) and 1× protease inhibitor cocktail (Sigma Chemicals, St. Louis, MO) by centrifugation (10,000 rpm in a 5415D Eppendorf™ centrifuge for 30 minutes at 4°C). The recovered volume from the sponge was calculated by subtracting the volume recovered from negative control sponges from the total recovered volume. Duplicate samples were pooled, frozen, and retrieved in batches for further analysis.

### Evaluation of HIV-1-specific and canarypox-specific antibody responses

Total HIV-1-specific immunoglobulin was quantified in plasma and rectal secretions at baseline as well as longitudinally post-immunization (Days 10, 17, 24, 180, and 365). Quantification of HIV-1-specific antibodies was performed with a modification of a previously described protocol [20] using the Vironostika® HIV-1 MICROELISA system (Organon Teknika Corp, Durham, NC). Samples were run according to the manufacturer's instructions with the addition of a standard curve generated using serial dilutions (10–3000 ng/ml) of human anti-HIV-1 gp120/160 IgG (Immuno Diagnostics, Inc.Woburn,MA). Total IgG and total IgA were quantified in the eluted rectal secretions or plasma by ELISA as previously reported [18], [19]. In brief, 96-well plates (Corning Inc., Corning, NY) were coated overnight at 4°C with rabbit anti-human IgG or IgA (Dako Corp, Carpenteria, CA) diluted 1/6000 in bicarbonate buffer (pH 9.6). Serially diluted standard curves utilized purified human immunoglobulin (IgG or IgA) ranging from 7.8–500 ng/ml (Jackson Immunoresearch Laboratories, West Grove, PA). Samples were run in duplicate, along with a positive control sample, for which performance characteristics and acceptable ranges had been previously established [21], [22]. Plates were incubated for 60 min at 37°C, and washed five times in wash buffer prior to the addition of 100 µl of peroxidase conjugated rabbit anti-human IgG or IgA (Dako Corp, Carpenteria, CA). Absorbance was read at 492 nm using a Benchmark Plus ELISA plate reader (Biorad, Hercules, CA) equipped with Microplate Manger® software. Values were expressed in ng/ml as extrapolated from standard curves, and the means were calculated for each sample. Final ELISA results were expressed in units of anti-HIV-1/µg of total IgG+IgA. Canarypox-specific antibodies in blood (IgG only) and rectal secretions (IgG+IgA) were detected by ELISA (supplies and protocol courtesy of Sanofi Pasteur) at the same time points.

### Isolation of mucosal mononuclear cells

Colonic mucosal mononuclear cells (MMC) were isolated from the sigmoid colon biopsies as previously reported [15], [17]. Briefly, biopsy samples were washed, collagenase digested, and disrupted into single cell suspensions in medium containing piperacillin-tazobactam antibiotic (Zosyn, Wyeth Co., Philadelphia, PA) and amphotericin B (Fungizone, GIBCO Invitrogen, Carlsbad, CA). This procedure routinely yielded between 2 to 5×10^6^ viable CD3^+^ T lymphocytes per 17 biopsies. Cell yield and phenotypes were quantified with Multi-Test staining and TRUCount beads (Becton Dickinson Immunocytometry Systems, San Jose, CA) respectively. The remaining biopsies were used for histology and tissue banking for later studies (IRB-approved).

### Polyclonal expansion of CD8^+^ T lymphocytes from PBMCs and MMCs

To obtain adequate numbers of CD8^+^ T lymphocytes (CTLs) for measurements of vaccine responses, CTLs from MMC and PBMC preparations were polyclonal expanded using a CD3:CD4 bi-specific monoclonal antibody as previously described [19]. Briefly, the cells were cultured for 14 days with the antibody (which inhibits CD4^+^ T lymphocyte growth and stimulates CD8^+^ T lymphocyte growth) plus IL-2 (with additional irradiated autologous feeder PBMC for MMC expansions). This procedure produces polyclonal expanded CTLs with minimal bias compared to non-expanded lymphocytes [19], [22]–[24]. Average yield of expanded CD3^+^ T lymphocytes was about 2×10^7^ expanded cells from 10^6^ fresh MMC [22]. Verification of expanded CTL numbers was performed using 3-color flow cytometry (CD3/CD4/CD8) and routinely demonstrated >85% purity of expanded CTLs from MMC and >95% from PBMC with a viability above 90% (data not shown).

### Evaluation of HIV-1-specific CD8^+^ T lymphocyte responses

Standard IFN-γ ELISpot assays were performed using bulk expanded CTLs as previously reported [21], [22], [24]. In brief, these cells were derived from MMC and PBMC [19] and then screened using a library of 15-mer peptides consecutively overlapping by 11 amino acids spanning the entire HIV-1 proteome sequence (NIH AIDS Research and Reference Reagent Repository catalogue numbers 8116, 6208, 9487, 5189, 5138, 6445, 6447, 6444, 6446), followed by reading with an automated ELISpot counting system (Cellular Technologies Limited, Cleveland, OH). Screening was performed against 53 pools of 12–16 consecutive peptides. Results for reactivity against peptide pools spanning protein sequences contained in the vaccine were expressed as spot-forming cells (SFC) per 10^6^ CTLs after background-subtracting the mean of the negative controls (consisting of peptide pools spanning protein sequences not contained in the vaccine, generally <50 SFC/well, usually <20 SFC/well). Baseline responses before treatment were established for every subject. These responses gave a false positive rate of 1.5%. The mean of the baseline responses was −5.5 SFC/10^6^ CTLs (95% CI −7.4 to −3.6).

### Statistical Analysis

Statistical analyses were carried out with Minitab® Statistical Software (State College, PA). The Wilcoxon Signed-Rank Test was used for comparisons of measurements from the same person across different time points. The Mann-Whitney test was utilized to compare groups of values, i.e. measurements from persons who received placebo versus vaccine, and measurements in the blood versus gut compartments. Note that in the blood versus gut compartment comparisons, both paired and non-paired non-parametric analyses were performed (to cover both possible assumptions regarding dependency between compartments) and yielded almost identical results. Statistical significance was defined as a *p* value for the null hypothesis of <0.05.

## Results

### Participant demographics

Twenty-two subjects enrolled in the study, of which three were found ineligible (out of range laboratory tests, initial non-disclosure of intravenous drug use, initial non-disclosure of HIV seropositivity) and one was withdrawn due to non-compliance; none of these received vaccinations. Eighteen study subjects including nine males and nine females (Table 1) were randomized to receive vaccine/placebo injections via either deltoid-IM or inguinal-SC injections. The median age was 39 years (range 25–60).

**Table 1:** Participant demographics.

		Subject	Age	Sex	Ethnicity
Placebo	Inguinal	H	42	F	African-American
		J	47	F	Caucasian
		U	60	F	Caucasian
	Deltoid	D	25	F	Caucasian
		K	45	F	African-American
		S	37	M	Caucasian
Vaccine	Inguinal	C	54	M	Caucasian
		F	55	F	African-American
		G	47	M	Caucasian
		M	26	M	Caucasian
		O	38	M	African-American
		Q	30	M	Caucasian
	Deltoid	B	38	F	Caucasian
		I	35	M	Caucasian
		N	25	M	Caucasian
		R	42	M	Asian-American
		T	29	F	Asian-American
		V	40	F	Caucasian

### Among vCP205 vaccinees, six of six tolerated deltoid intramuscular vaccinations, and four of six tolerated inguinal subcutaneous vaccinations

All 18 subjects completed all protocol visits, although 2/18 in the inguinal vaccine group (Subjects C and M) had adverse events (AEs) at the injection sites after the 2nd vaccination and did not receive subsequent vaccinations. Among placebo vaccinees, all AEs in both deltoid and inguinal groups were mild (grade 1 or 2). Among the six deltoid-IM vaccinees, there were 31 grade 1, 3 grade 2, and no grade 3 or 4 AEs. Among the six inguinal-SC vaccinees, there were 29 grade 1, 5 grade 2, 3 grade 3, and no grade 4 AEs. All grade 3 AEs were in the same individual receiving vaccine (Subject M), who had swelling, tenderness, and erythema at the injection site. Of the six inguinal-SC vaccinees, Subjects C and M halted vaccinations due to injection site inflammation after the second vaccination; the symptoms resolved spontaneously and these two subjects completed the full monitoring and sample collection protocol. Thus, in contrast to deltoid-IM vaccination with vCP205, inguinal-SC vaccination was not entirely safe.

One subject (Subject S) in the deltoid-IM placebo group had true HIV-1 infection detected only at the final study visit (Day 365) demonstrating 9,870 copies/ml of plasma HIV-1 RNA, and reactive serum anti-HIV antibodies confirmed by Western blot including reactivity against non-vaccine HIV-1 proteins. All HIV testing at the prior study visit (Day 180) had been negative.

### All vaccinees had humoral responses against the canarypox vector in blood but not in rectal mucosa

**Figure 2:**
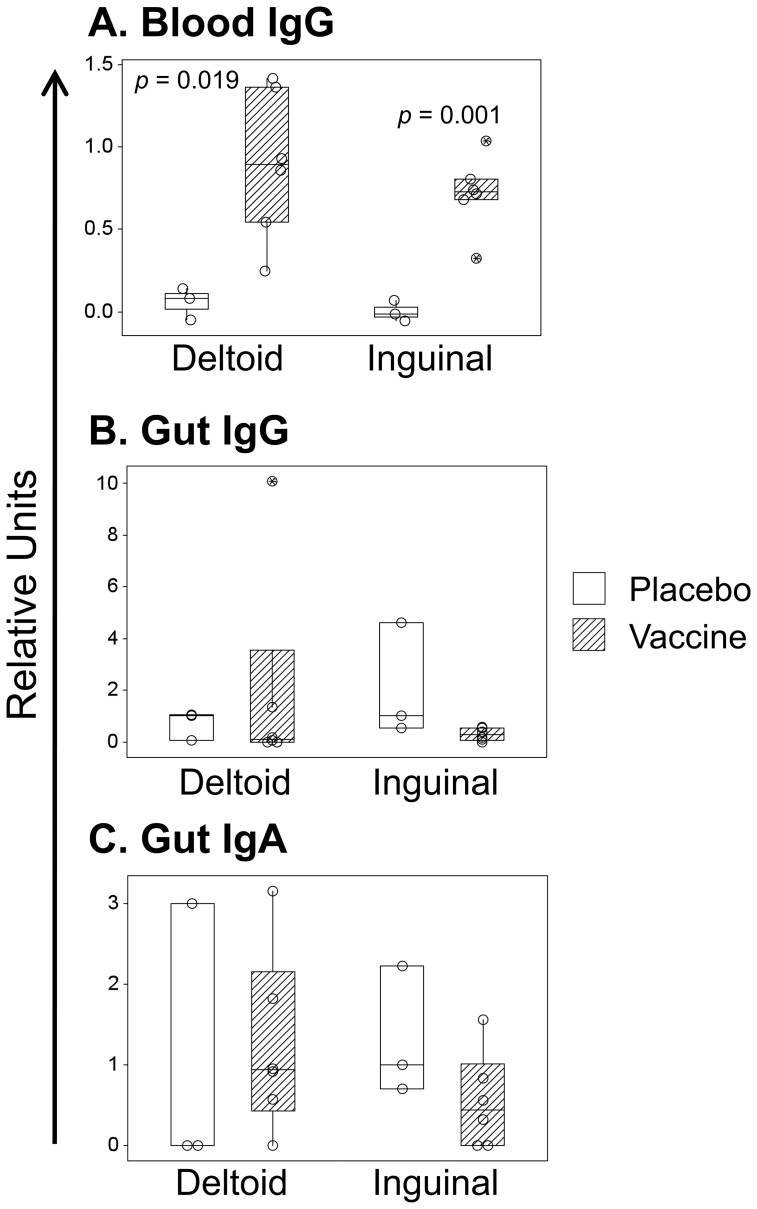
Mucosal and blood antibody responses against Canarypox. Anti-Canarypox antibodies were measured by ELISA in blood (IgG only, Panel A) and gut mucosal secretions (IgG and IgA, Panels B and C) on Day 24. Responses against placebo or vCP205 vaccine are plotted by vaccination route, as abstract units (normalized against placebo and background-subtracted). Medians and 95% confidence intervals are indicated for each group.

The 12 vaccinees were assessed for their canarypox-specific antibody responses in blood plasma (IgG) and gut secretions (IgG and IgA) three days after the fourth weekly immunization (Day 24). Blood (Figure 2a) demonstrated significant anti-canarypox responses for both deltoid (*p* = 0.019) and inguinal (*p* = 0.001) groups. In contrast, there were no statistically significant IgG or IgA responses against canarypox in the gut (Figure 2b), although there was an increase for IgA in deltoid vaccinees that did not reach statistical significance. Overall, there were no significant differences in canarypox humoral responses for deltoid versus inguinal vaccination.

### HIV-1-specific antibodies developed slowly in the gut and remained essentially undetectable in the blood

HIV-1-specific blood and gut mucosal antibody responses were longitudinally assessed over the 365 days after first immunization (Table 2). In blood, only one vaccinee (Subject B) had detectable HIV-1-specific antibodies. Gut mucosal responses were observed on Day 180 when 2/9 (22%) vaccinees had detectable HIV-1-specific antibodies (0/4 deltoid and 2/5 inguinal vaccinees). This increased on Day 365 to 3/9 (33%) of evaluated vaccinees (2/4 deltoid and 1/5 inguinal vaccinees). Only 1 participant (Subject F) demonstrated repeated antibody responses on Days 180 and 365, and only in the gut. Placebo recipients had no HIV-1-specific antibodies at any time point, except for one person who actually sustained HIV-1 infection between Days 180 and 365 (Subject S). As a whole, these data demonstrate that detectable humoral responses against the HIV-1 portion of the vaccine appeared only in the gut, not blood, and were observed late (after 24 days, up to 180 days or later).

**Table 2:** Vaccine-induced antibody responses against HIV-1.

		Day:	0		10		17		24		180		365	
			*Gut*	*Blood*	*Gut*	*Blood*	*Gut*	*Blood*	*Gut*	*Blood*	*Gut*	*Blood*	*Gut*	*Blood*
**Placebo**	**Inguinal**	**H**	-	-	-	-	ND	-	-	-	-	-	-	-
		**J**	-	-	-	-	ND	-	-	-	-	-	-	-
		**U**	-	-	-	-	ND	-	-	-	-	-	-	-
	**Deltoid**	**D**	-	-	-	-	ND	-	-	-	-	-	-	-
		**K**	-	-	-	-	ND	-	-	-	ND	ND	-	-
**Vaccine**	**Inguinal**	**C**	-	-	-	-	ND	-	-	-	-	-	-	-
		**F**	-	-	-	-	ND	-	-	-	**+**	-	**+**	-
		**G**	-	-	-	-	ND	-	-	-	ND	ND	ND	ND
		**M**	-	-	-	-	ND	-	-	-	-	-	-	-
		**O**	-	-	-	-	ND	-	-	-	-	-	-	-
		**Q**	-	-	-	-	ND	-	-	-	**+**	-	-	ND
	**Deltoid**	**B**	-	-	-	-	ND	-	-	-	-	-	**+**	**+**
		**I**	-	-	-	-	ND	-	-	-	-	-	-	-
		**N**	-	-	-	-	ND	-	-	-	ND	ND	ND	ND
		**R**	-	-	-	-	ND	-	-	-	-	-	-	-
		**T**	-	-	-	-	ND	-	-	-	-	-	**+**	-
		**V**	-	-	-	-	ND	-	-	-	ND	ND	ND	ND

“-”: below limits of detection

ND: sample not done.

### Inguinal immunization induced HIV-1-specific CTL responses in both blood and gut

**Figure 3:**
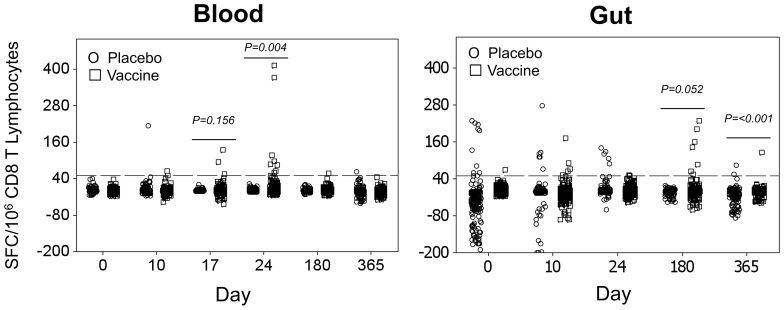
Total observed CTL responses against HIV-1 in blood and gut mucosal compartments. The background-subtracted CTL responses of all participants against HIV-1 vaccine peptide pools were summed for persons who received placebo (circles) and vCP205 vaccinations (squares) and plotted for blood (left) and gut mucosa (right). *p*-values indicate significant differences between groups by Students t-test.

The two vaccination groups were compared for CTL responses in both blood and gut mucosa. On Days 0, 10, 17, 24, 180, and 365 after the first vaccination, HIV-1-specific CTL responses were assessed in both compartments by IFN-γ ELISpot assay for reactivity against the HIV-1 protein sequences expressed by vCP205. Baseline responses before treatment were established for each subject in both compartments. The mean of the baseline background-subtracted responses was −5.51 (95% CI −7.42 to −3.62) spot-forming cells per million CD8^+^ T lymphocytes, with a false positive rate of 1.5%. In blood (Figure 3a), there was a significant increase in HIV-1-reactivity (*p* = 0.004) by Day 24. For gut (Figure 3b), the response was borderline significant on Day 180 (*p* = 0.052) and significant on Day 365 (*p*<0.001).

**Figure 4:**
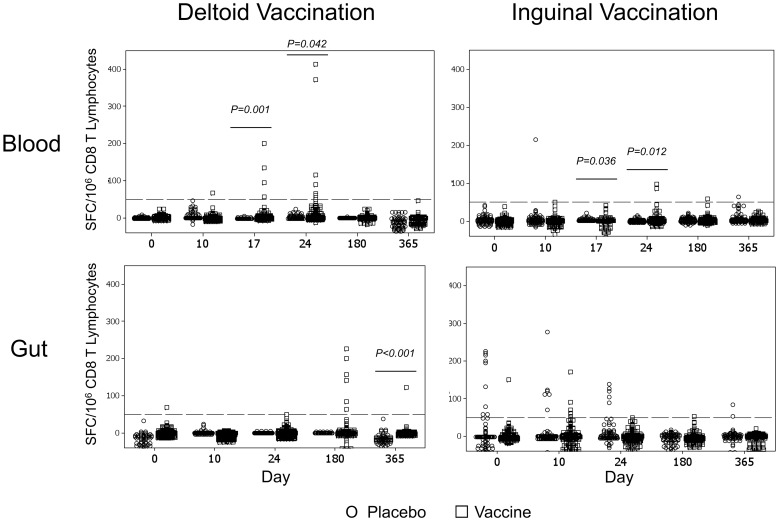
HIV-1-specific CTL responses in blood and gut mucosal compartments divided according to vaccination route. The results in Figure 3 are plotted separate depending on vaccination route for recipients of saline (circles) and vCP205 vaccine (squares). The upper and lower panels show blood and gut mucosal responses respectively, and the left and right panels give results for persons who received deltoid and inguinal vaccinations respectively. *p*-values indicate significant differences between groups by Students t-test.

Across groups, there appeared to be compartment-specific differences in HIV-1-specific CTL responses based on vaccination route. In blood (Figure 4 top panels) the timing was similar for both vaccination routes, achieving significance by Day 17 (*p* = 0.001 and *p* = 0.036 for deltoid-IM and inguinal-SC vaccinees, respectively) and Day 24 (*p* = 0.042 and *p* = 0.012). There was a suggestion that blood responses were higher in magnitude on Day 24 in the deltoid versus inguinal group (mean 167 versus 91, *p* = 0.21). In gut mucosa (Figure 4 bottom panels), however, only the deltoid vaccination group achieved significant responses and then only on Day 365 (*p*<0.001), although a non-significant increase was observed on Day 180. There were several early gut mucosal responses in inguinal vaccinees, but these did not reach significance across the group. Overall, these analyses of pooled group data suggest that deltoid vaccination may induce higher magnitude CTL responses in blood than inguinal vaccination at the early time points examined, and that there may be kinetic differences in the different compartments varying by vaccination route.

### HIV-1-specific CTL responses were generated earlier in blood than gut

Examining HIV-1-specific CTL responses within individual vaccinees, defined as interferon-γ ELISpot measurements of ≥50 spot-forming cells per million CD8^+^ T lymphocytes, responses in blood and gut mucosa displayed different kinetics.

**Figure 5:**
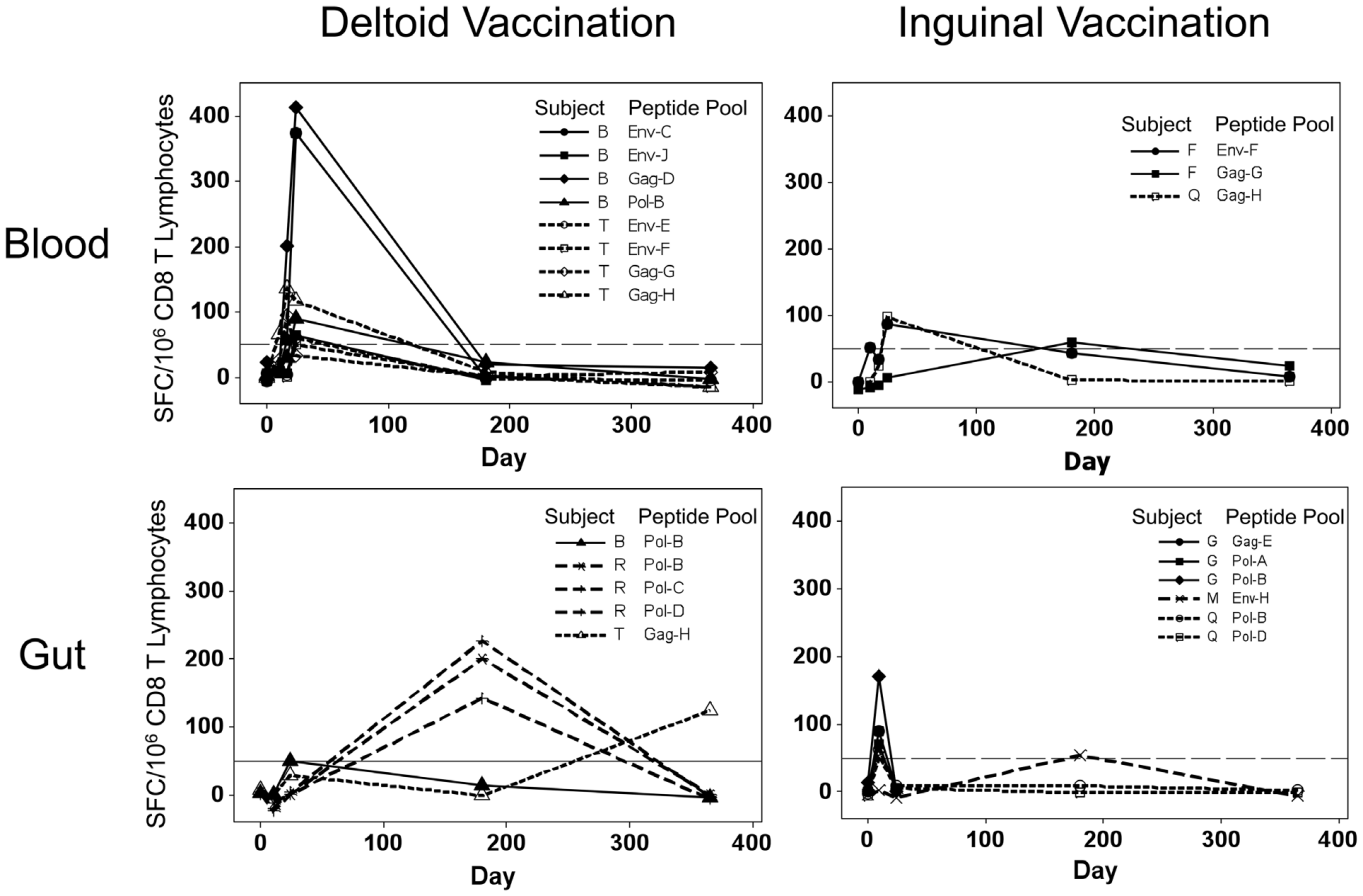
Kinetics of HIV-1-specific CTL responses within individuals. CTL responses that achieved a magnitude of ≥50 spot-forming cells per million CD8^+^ T lymphocytes at any time are plotted. The upper and lower panels show blood and gut mucosal responses respectively, and the left and right panels give results for persons who received deltoid and inguinal vaccinations respectively.

By this criterion, 4/12 (33%) vaccinees had detectable blood responses, including two from each vaccination group (Figure 5 top panels). The deltoid vaccination responders appeared to have higher magnitude and breadth of responses compared to inguinal vaccinees at the tested time points, consistent with the overall group comparisons. The two deltoid vaccine responders recognized 4 peptide pools per person, whereas the two inguinal vaccine responders recognized 1 and 2 pools. Both groups had detectable CTL responses within 24 days after vaccination initiation.

Within gut mucosa, 6/12 (50%) vaccinees had CTL responses, including three from each vaccination group (Figure 5 bottom panels). In contrast to the blood, the kinetics of responses appeared different between the groups. The deltoid vaccine responders had highest magnitudes observed at Day 180, while the inguinal vaccine responders had highest magnitudes on Day 17 (*p* = 0.0357 by Chi square test). Also in contrast to blood, the breadth of CTL responses was similar between groups, ranging from 1 to 3 peptide pools for each individual. These data suggest that the route of vaccination protocol influences the kinetics and magnitude of HIV-1-specific responses in blood and gut mucosal compartments, with deltoid vaccination eliciting higher magnitude and broader responses in the blood and delayed responses in the gut mucosa compared to inguinal vaccination, for the time points tested.

### CTL targeting of HIV-1 was discordant between blood and gut compartments within individuals and affected by vaccination route

**Figure 6:**
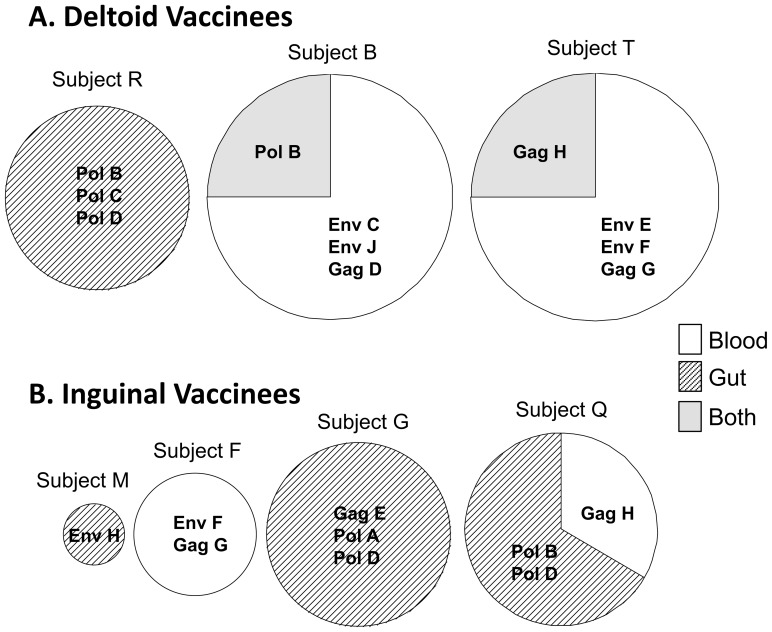
Overlap of HIV-1-specific CTL responses between blood and gut mucosal compartments. For each of the indicated participants with detected HIV-1-specific CTL responses, a pie chart indicates the fraction of peptide pool responses that were detected in blood only, gut mucosa only, or both compartments. The size of the circle is proportional to the number of peptide pool responses.

CTL responses against peptide pools were compared between blood and gut in each responder (Figure 6). One deltoid vaccinee (Subject R) displayed responses to 3 pools in the gut only. The other two deltoid vaccinees (Subjects B and T) each had 3 responses only in the blood, one concordant response in blood and gut, and no responses in gut alone. Three of the inguinal vaccinees (Subjects G, Q, and M) had a predominance of responses in the gut only, and the fourth (Subject F) had responses in the blood only; none had concordant CTL responses in both compartments. Note that because these are measurements with peptide pools, concordance of CTL responses against peptide pools may overestimate concordance of recognized epitopes. Overall, however, these results suggest that deltoid vaccination preferentially induces CTL responses in blood with some concordance in gut mucosa, while inguinal vaccination tends to induce more responses only in the gut mucosal compartment at the time points evaluated.

## Discussion

Despite the role of mucosal surfaces in sexual transmission of HIV-1 and the central involvement of the gut in the pathogenesis of acute and chronic infection, data regarding vaccine responses in the human gut mucosa are lacking. To date, no large scale clinical HIV-1 vaccine trial has evaluated immunity in this compartment, and only one vaccine has demonstrated any hint of clinical efficacy. This vaccine, tested in the RV144 trial, was a prime-boost combination of recombinant canarypox (vCP1521) and gp120 subunit (AIDSVax) vaccines, each of which failed to produce their intended cellular and humoral immune responses when tested individually. In this study, we utilize vCP205 (based on the same vector as vCP1521), and test it in an FDA Phase I trial for capability to elicit gut mucosal immune responses when delivered in an intensive regimen of 4 weekly administrations, and evaluate whether inguinal vaccination might augment vaccine-specific immune responses in the gut.

Past macaque data indicate that inguinal vaccination can boost mucosal immune responses in comparison to standard intramuscular immunizations [11], and our trial evaluated the clinical feasibility and mucosal immunogenicity of this approach. The data indicated that the protocol is safe and well tolerated by the volunteers, similar to our earlier small study examining inguinal versus deltoid vaccination with a recombinant vaccinia virus HIV-1 vaccine [20]. In general, the inguinal subcutaneous vaccination route was safe and well tolerated, with only minor localized injection site symptoms.

Evaluation of humoral immunity showed a discrepancy between responses to the vector versus its HIV-1 inserts, likely related to the relatively large proteome of the canarypox vector versus the HIV-1 inserts, without regard to route of vaccination. After vaccination, antibodies recognizing canarypox could be detected in the blood at the Day 24 time point (two days after the last vaccination), but HIV-1-specific antibodies were not detectable at that time, and seen only at the next time points of 180 or 365 days in 4/9 tested individuals. Titers of these antibodies in gut mucosal secretions were far below those seen in HIV-1-infected persons [20], and appeared to wane in Subject Q. The requirement of several months to generate these responses was unexpected, but the data highlight the compartmentalized nature of blood versus gut mucosal immunity. Our low blood HIV-1 humoral response rate is not inconsistent with the generally low responses detected in blood in trials of recombinant canarypox vaccines without heterologous priming or boosting [25], [26], and may be even lower due to the short term vaccination in our study (Days 0, 7, 14, 21) versus the usually prolonged regimens in other studies (Months 0, 1, 3, 6, ±9, ±12).

While vCP205 vaccine was designed to generate HIV-1-specific CTL responses, it was found to be weakly immunogenic for HIV-1-specific CTLs in prior clinical studies [12], [25], [27]. Our data demonstrated a blood response rate of 4/12 (33%), similar to the earlier trials of this vaccine, and a gut mucosal response rate of 6/12 (50%) overall. Although response rates appeared similar for deltoid versus inguinal vaccination, there appeared to be a difference in the kinetics of the responses. Inguinal vaccination resulted in earlier gut mucosal responses than deltoid vaccination, suggesting that the closer anatomic proximity of injection yielded more direct access.

Our data also hinted at compartmentalization of CTL responses between blood and gut mucosa. Of the seven CTL responders (Subjects B, F, G, M, R, T, and Q), three had responses in both compartments (B, T, and Q), one had responses in the blood only (F), and three had responses in the gut mucosal compartment only (G, M, and R). For persons targeting both compartments, CTL targeting demonstrated distinct profiles. The highest magnitude responses against peptide pools in each compartment were not observed in the other compartment, which indicated that this was not an artefact of the limit of detection. It is unclear whether these results reflected bias due to weak immunogenicity of the vaccine, in which case a strongly immunogenic vaccine might give concordant results in both compartments, as we have observed for HIV-1 infection [22] and others have observed with recombinant adenovirus vaccination of macaques [28], [29]. Still, the data do suggest that the route of immunization affected the quantity of antigenic access to the two compartments. The timing of sampling was based on anticipation that peak responses would occur soon after the final vaccination, but surprisingly our assessments likely missed peak responses between 24 and 180 days, rendering comparisons of peak magnitude and breadth of CTL responses unreliable. Still, there were observed differences at the evaluated time points, indicating at least differences in the kinetics of immune responses.

A potentially important difference between our vaccination protocol and prior macaque inguinal vaccination data showing better access to the mucosa [11] was the limitation of our inguinal vaccination to subcutaneous tissue, compared to deep inguinal vaccinations performed in macaques, prompted by safety concerns. Still, our results suggested that even subcutaneous inguinal vaccination might better access the lower gut mucosal immune compartment, although deltoid intramuscular vaccination also showed mucosal access, perhaps delayed because those CTLs trafficked from the periphery. Anatomically, superficial inguinal lymph nodes drain through muscle and skin, whereas deep inguinal lymph nodes share drainage with intra-abdominal structures. Animal data suggest that direct mucosal vaccination is superior for generating mucosal immune responses [5], [10], but it is unclear whether a replication defective vector would achieve enough immunogenicity without mucosal injection, which would be clinically difficult in humans.

In conclusion, this HIV-1 vaccine demonstrated differential immunogenicity for blood and gut mucosal compartments. The kinetics and targeting of humoral and CTL responses varied considerably between these compartments, and there was a surprising lag in gut mucosal responses after deltoid vaccination. Our results highlight a potential importance of route of vaccine administration, and also indicate that short term measurements of immune responses in the blood are unreliable for assessment of mucosal immunity from HIV-1 vaccine candidates.

## Supporting Information

Protocol S1 **Detailed vaccine study protocol.**
(DOC)

Checklist S1 **CONSORT checklist for study.**
(DOC)
