# Supplementary material for: Differential Blood and Mucosal Immune Responses against an HIV-1 Vaccine Administered via Inguinal or Deltoid Injection
Source: PLoS One. 2014 Feb 18;9(2):e88621. doi: 10.1371/journal.pone.0088621 (PMC3928250; doi:10.1371/journal.pone.0088621)
Supplement: Protocol S1 — Detailed vaccine study protocol. (DOC) [file pone.0088621.s001.doc]

### Protocol

### A Phase 1 double blind placebo controlled trial to evaluate the safety and immunogenicity of the Aventis Pasteur ALVAC-HIV (vCP205) administered to the groin area versus the deltoid area

### Vaccine Provided by

**Aventis Pasteur S.A.**

**Clinical Site Data Coordinator**

##### David Geffen School of Medicine at UCLA UCLA School of Medicine

## Peter A. Anton, M.D., Principal Investigator

## TABLE OF CONTENTS

Page
 Protocol Summary iii

Contact List of Investigators iv

Investigator Qualifications v

Name and Address of Research Facilities to be Used xvi

Name and Address of Reviewing Institutional Review Board xvii

1.0 Objectives and Purpose of Study 1

2.0 Introduction and Rationale 2

3.0 Product Overview 3

4.0 Experimental Design 6

5.0 Data and Safety Monitoring 6

6.0 Statistical Analysis 8

7.0 Randomization and Blinding 9

8.0 Human Subjects 9

9.0 Immunization Procedures 13

10.0 Immunologic and Virologic Studies 14

11.0 Literature Cited 27

Appendices

Consent Form Appendix 1

Case Report Form Appendix 2

Risk Reduction Patient Handout Appendix 3

Daily Diary Appendix 4

Adverse Experiences Report Form Appendix 5

Severity Grading Guide for Adverse Experiences Appendix 6

**PROTOCOL SUMMARY**

**Protocol 003:** Randomized Double Blind Placebo Controlled Trial to Evaluate the Safety and lmmunogenicity of the Aventis Pasteur ALVAC-HIV (vCP205) Administered to the Groin Area versus the Deltoid Area

**Subjects:** Healthy adult subjects who have no identifiable higher risk behavior for HIV-1 infection.

**Number of Subjects:** 18 completing all visits to day 24 (completion of all baseline, vaccination, safety and immediate immune response visits) - based in past experience we will “over-enroll” (signing consent) up to a total of 24 to allow for subject or investigator initiated withdrawal.

Schema:

| Route/Volume of ALVAC-HIV (vCP205) or placebo | IMMUNIZATIONS | | | |
| --- | --- | --- | --- | --- |
|  | 1 (day 0) | 2 (day 7) | 3 (day 14) | 4(day 21) |
| Subcutaneous (groin)  or  Intramuscular (deltoid)  1 ml | ALVAC-HIV (vCP205) | ALVAC-HIV (vCP205) | ALVAC-HIV (vCP205) | ALVAC-HIV (vCP205) |
|  | placebo | placebo | placebo | placebo |

**Study period for each subject:** 14 months (starting at day –42 through week 52)

**Follow up period for each subject:** 26 months (includes 1 year of as needed subject-initiated visits and PCR testing)

Time period for trial: 2 years

**Safety Monitor:** Ronald Mitsuyasu, M.D

Director, Center for Clinical AIDS Research and Education (CARE)

UCLA, School of Medicine

**Funding Agency:** National Institute of Allergy and Infectious Diseases

**Clinical Site:** University of California, Los Angeles

**Data Coordination:** University of California, Los Angeles

CONTACT LIST FOR INVESTIGATORS

UCLA School of Medicine

Center for HIV and Digestive Diseases

675 Charles E. Young Drive South

2734 MacDonald Research Laboratories

Los Angeles, CA 90095-7019

Peter Anton, M.D. (310) 206-5797

Cellular Immunology and Cytometry Laboratory

10833 Le Conte Avenue

12-240 Factor Bldg.

Los Angeles, CA 90095-1745

Beth Jamieson, Ph.D. (310) 206-8217

UCLA Center for Clinical AIDS Research and Education (CARE)

Room BH-412 CHS

10833 Le Conte Avenue

Los Angeles, CA 90095-1793

Ronald Mitsuyasu, M.D. (310) 206-8359

Aventis Pasteur S.A.

Discovery Drive

Swiftwater, PA 18370

Sanjay Gurunathan, M.D. (570) 839-6185

**INVESTIGATORS QUALIFICATIONS**

NIH biosketches of the investigators involved in this project are included on the following pages to document their medical and scientific expertise. Their specific roles on the project are described here.

Peter Anton, M.D., a gastroenterologist, is an Associate Professor in the UCLA Department of Medicine and Director of the UCLA Center for HIV and Digestive Disorders. He is the PI of the IND to the FDA and will administer the vaccines, obtain mucosal biopsies and be responsible for patient care. He will be responsible for reporting and managing any adverse events resulting from the study and maintaining appropriate follow-up.

Beth Jamieson, Ph.D., a cellular immunologist, is an Assistant Professor in the UCLA Department of Medicine and Director of the UCLA Cellular Immunology and Cytometry Laboratory. She will design and oversee the immunologic assays used to determine the immunogenicity of the vaccine inoculations. This will insure using established immunological assays as well as development of mucosal adaptations to assess local immune response.

Otto Yang, M.D. is an Assistant Professor of Medicine in the Division of Infectious Diseases, Department of Medicine, UCLA School of Medicine. Dr. Yang’s laboratory will perform the non-specific and antigen specific expansion of isolated mucosal mononuclear cells, characterizing these cell types by flow cytometry and direct the epitope mapping to demonstrate the immunogenicity of the candidate vaccines.

Ronald T. Mitsuyasu, M.D., a hematologist, is a Professor in the UCLA Department of Medicine and Director of the UCLA Clinical AIDS Research and Education Center. He will oversee the safety monitoring for this study.

## NAME AND ADDRESS OF RESEARCH FACILITIES TO BE USED

UCLA School of Medicine

Cellular Immunology and Cytometry Laboratory

10833 Le Conte Avenue

12-240 Factor Building

Los Angeles, CA 90095-1745

and

Center for HIV and Digestive Diseases

675 Charles E. Young Drive South

2734 MacDonald Research Laboratory

Los Angeles, CA 90095-7019

**NAME AND ADDRESS OF REVIEWING INSTITUTIONAL REVIEW BOARD**

Office for Protection of Research Subjects

UCLA Medical Institutional Review Board

2107 Ueberroth Building

Los Angeles, CA 90095-1694

1. **OBJECTIVES AND PURPOSE OF STUDY**

The goal of this vaccination study, using a randomized double-blind placebo controlled methodology, is to evaluate the safety and immunogenicity of ALVAC-HIV (vCP205) which contains the following inserted HIV-1 genes: the glycoprotein regions encoding gp120 (HIV-1_MN_) linked to the membrane-spanning portion of gp41 (HIV-1_LA1_), the genes encoding the entire Gag protein, and a portion of the Pol sequence (HIV-1_LA1_), sufficient to encode protease activity. The recombinant vaccine construct was developed by Virogenetics Corporation, Troy, New York. This experimental vaccine is manufactured by Aventis Pasteur S.A., Marcy L´Etoile, France. This experimental vaccine will be administered by subcutaneous injection in the groin (6 subjects), an area that drains to the inguinal lymph nodes to stimulate immunologic activity (locally) in the draining rectal mucosa as well as in the blood (systemically). Six subjects will receive the experimental vaccine intramuscularly into the deltoid region and will serve as the comparator arm of the study. In addition 6 subjects will receive placebo (3 groin and 3 intramuscularly)

**1.1 Primary Objectives**

To evaluate the safety of administering ALVAC-HIV (vCP205) vaccinations to healthy adult individuals using a subcutaneous targeted inguinal lymph node (TILN) or deltoid intramuscular vaccination at 1 x 10^6.87^ TCID_50_ of ALVAC-HIV (VCP205) (Days 0,7,14 & 21)

**1.2 Secondary Objectives**

To compare the immunogenicity of subcutaneous (SQ) vaccination with four doses of ALVAC-HIV (vCP205) administered in the groin area versus four doses administered intramuscularly (IM) in the deltoid region versus four doses of placebo administered IM in the deltoid region or SQ in the groin area by determining whether successive immunizations induce increased levels of one or more of the following:

### CTL activity directed to canarypox and HIV-1 *env*, *gag* and a portion of the *pol–*gene products

### Anti-HIV-1 directed CD4^+^ T cell proliferative response to soluble p24 antigen

### CD8^+^ T cell specificity for HIV-1 epitopes

### Serum and mucosal immunoglobulins against HIV-1 epitopes

### Non-specific indicators of immune activation: β-chemokine production, HLA-DR and CD38 on CD8 cells from blood and gut

1. **INTRODUCTION AND RATIONALE**

Human immunodeficiency virus type 1 (HIV-1) infection/AIDS has emerged as a worldwide public health problem associated with extensive morbidity and mortality. Although recent advances have been made in antiretroviral therapy against HIV-1, there is currently no cure. Ultimate control of the disease will likely depend on the development of a safe and effective vaccine against HIV-1.

HIV-1 is primarily a sexually transmitted disease^1^ that as of November 2002 has infected at least 42 million people worldwide since the beginning of the epidemic and at least 5 million people are infected annually^2^. It is most commonly transmitted via a mucosal surface. The mucosal lining itself is a primary potential site of both humoral and cellular protection consisting of B lymphocytes, activated memory T lymphocytes, secretory IgA, a host of antigen presenting cells (APC) including an extensive network of mucosal dendritic cells and baseline elevated levels of β-chemokine expression^3^. The normal state of activity in the gastrointestinal mucosa is one of mild, controlled inflammation, which consequently provides increased viral targets. A protective vaccine will likely need to induce immune responses at the mucosal surfaces that are the portals of entry of HIV-1 into the body. Ideally, the vaccine would also induce systemic immunity so that virus that escapes neutralization by the mucosal immune response could be attacked by barriers of T lymphocytes and antibodies present in lymph nodes and blood.

The animal model that best mirrors human HIV-1 disease is simian immunodeficiency virus (SIV) infection of rhesus macaques. Previous work on mucosal immunity to SIV in the rhesus model has guided the development of the current protocol. The current protocol is designed to reproduce in humans knowledge learned from the SIV model, in particular, that delivery of immunogen to the groin area can confer protection against SIV infection in a sexual transmission model, while the same immunogen delivered by other routes fails to protect. Total protection against transmission of SIV across the rectal mucosa was achieved in 4 of 7 macaques and partial protection was achieved in the remaining 3 of 7 when immunogen was delivered by targeted iliac lymph node immunizations. Infection occurred in 13 of 14 unimmunized macaques or those immunized by other routes^4^.

The targeted lymph node route of immunization involves vaccine injection into the subcutaneous tissue near a lymph node. This efficiently uses the host’s antigen presenting cells to deliver antigen to the lymph node where specific immune responses develop that subsequently may protect the host from infection with the pathogen. The iliac and inguinal lymph nodes are the primary draining lymph nodes of the genitourinary and rectal tracts ^5,6^. Therefore, this protocol will use groin immunizations (SQ, Inguinal region) as the site for delivery of immunizing antigen to nodes draining the rectal mucosa. As the axillary and supraclavicular lymph nodes are those primarily draining the deltoid area and these nodes do not drain rectal mucosal surfaces, their associated non-mucosal drainage region (IM deltoid) will be used as a comparative immunization site/route. No sampling of nodes is planned in this study.

In the SIV/macaque model, protection against rectal challenge was associated with a significant increase of sIgA-secreting cells in the lymph nodes and of sIgA at the mucosal surface, and increases in CTL activity, CD4^+^ specific T cell proliferative responses to *gag* proteins and CD8-suppressor factor and β-chemokine production from blood mononuclear cells and autopsy mucosa. The immunization thus provoked immune responses on three levels of immunity: mucosal, in draining lymph nodes, and in the circulation. These responses may all contribute to more complete protection of the host against viral challenge. While secretory IgA and β-chemokines operate at the mucosal surfaces, in the lymph nodes or circulation, alternate mechanisms including CTL activity may play the pivotal role if the mucosal barrier is breached.

In our protocol, the routes (subcutaneous and IM) of administration of the vaccine has been previously used in prior ALVAC-based vaccine trial subjects^7^ only the site (groin area) is novel. This site was selected in order to stimulate an immune response in the inguinal nodes, which serve as an inductive site to sensitize lymphocytes that home to the mucosa. The route of immunization typically used for vaccines, i.e., immunization in the skin of the arm, is associated with induction of systemic immunity (i.e., immunity detectable in the blood) but has not been evaluated for induction of mucosal immune responses. This is because the inductive sites for mucosal immune responses are separate from those for systemic immunity. Evidence that the site of immunization is critical for protection against transmission of HIV-1/SIV across at least the rectal mucosa is provided by the results of a previous study in monkeys where immunization by conventional routes did not confer protection against rectal transmission, while immunization by targeted lymph node immunization in the groin area did confer protection^4^.

One hundred and fifty-one non-ALVAC-HIV (vCP205) inoculations into the groin area of 37 macaques have been done in the course of Dr. Thomas Lehner’s studies using combinations of immunogens and/or adjuvants with no adverse events. Thus, groin immunization itself is thought to be safe and there is no reason to expect that ALVAC-HIV (vCP205) inoculation into this site in humans, rather than the arm would cause specific safety concerns.

Building on prior protective immunizations in the non-human primate model following targeted lymph node immunizations, we will determine whether a similar HIV-1 immunization strategy in humans elicits potentially protective immune responses in the blood and in the rectal mucosa. By targeting our vaccine to the mucosal immune system, we expect to successfully stimulate a detectable memory CD8^+^ T cell response as well as local immunoglobulin production in all of our subjects. This, coupled with the primary endpoints of safety, would represent desirable endpoints for this Phase I study.

**3.0 PRODUCT OVERVIEW**

**3.1 Background and rationale**

Early studies in adult volunteers evaluating live recombinants were based on vaccinia virus that expressed HIV envelope (gp160) antigen. These studies demonstrated that priming vaccinia-naive volunteers twice by scarification with a live vaccinia virus-gp160 recombinant (HIVAC-1e, Oncogen/Bristol Myers Squibb Inc.) elicited CD8+ CTL activity in 45% of the vaccines. Smallpox vaccination with standard vaccinia has been generally regarded as safe, however, serious post-vaccinia complications including fatal vaccinia necrosum (progressive vaccinia) in immunocompromised persons, encephalitis, disseminated vaccinia, and autoinoculation of the eyes have been observed occasionally. Enthusiasm for wide-scale use of a recombinant vaccinia virus vaccine for prevention and control of HIV infection has been tempered by issues of safety and the possibility of transmission of vaccinia virus to close contacts. To circumvent these safety issues, an avian poxvirus vector (canarypox virus) satisfies many of the criteria for an HIV vaccine. Like vaccinia virus, canarypox can accommodate large amounts of foreign DNA in its genome, infect mammalian cells and cause them to produce foreign proteins and is thermostable at ambient temperatures. In contrast to vaccinia virus, canarypox virus is host-range restricted. In mammalian cells, it undergoes an abortive cycle of replication and does not produce infectious progeny virus. Intracellular processing of canarypox-expressed foreign HIV proteins via the MHC Class I pathway leads to induction of cytotoxicT-lymphocyte activity. High doses of canarypox virus have not caused adverse effects in a wide variety of animals, even in profoundly immunosuppressed animals. This means that canarypox recombinants cannot disseminate and cause progressive disease in human recipients or be transmitted to unvaccinated contacts. Canarypox vectors have been safely administered to thousands of volunteers, including HIV-infected persons.

##### 3.2 Human experience with ALVAC vectors

As of Nov-2002, ALVAC candidate vaccines have been administered to over 2000 HIV seronegative individuals expressing proteins from rabies (N=285), measles (N=65), CMV (N=70), Japanese encephalitis (N=12), or HIV (N>1800). Five different ALVAC-HIV vaccine constructs have been studied in over 20 placebo-controlled, double blind trials. These studies have been conducted in the Americas, EU, Africa and Thailand and have involved various HIV risk strata and age groups, including infants. ALVAC has been administered in combination with subunits (rgp160, rgp120), p24, Remune™, DNA vaccines, and to vaccinia primed volunteers*.* In addition ALVAC-HIV vaccines have been used in over 300 HIV-1 infected volunteers without significant safety concerns ^8^.

The recombinant canarypox virus to be used in this study, ALVAC-HIV (vCP205), expresses the products of multiple HIV-1 genes as follows: the gag gene expressing the gag p55 protein of the HIV-1 LAI strain, the protease portion of the pol gene, expressing the p15 protein of the HIV-1 LAI strain, a part of the env gene expressing gp120 of the HIV-1 MN strain, and the anchoring transmembrane region of gp41 glycoprotein of the HIV-1 LAI strain.

ALVAC-HIV (vCP205), has been administered to over 600 healthy human volunteers including infants. A review of the available human experience to date suggests that ALVAC-HIV vaccines are well tolerated and acceptable to volunteers*.* In a study by Belshe, et al. describing the safety and immunogenicity of ALVAC-HIV (vCP205) among ~420 high and low risk HIV seronegative volunteers, the majority of the solicited local and systemic reactions were mild to moderate in intensity^9^. Six and 9 volunteers reported severe local and systemic reactions respectively*.* None of the volunteers had both severe systemic and local reactions or were excluded from additional vaccinations*.* Moreover, no increase in local and systemic reactions was seen with repetitive dosing*.* Several studies have evaluated various ALVAC-HIV (vCP205) schedules and doses*.* These studies suggest that multiple administrations of ALVAC-HIV (vCP205) are needed for adequate induction of CTL responses and the number of doses administered is a significant predictor of the CTL response rate ^10^. The net cumulative CTL response rates attributable to vaccination in these studies ranged from 30 to 60% ^9,10,11^*.* Importantly, CD8+ T cells derived from recipients of ALVAC-HIV (vCP205) vaccine were capable of lysing autologous CD4+ lymphoblasts infected with primary HIV-1 isolates representing genetically diverse viral clades demonstrating a broad pattern of cytolysis and cross reactivity ^12^.

Please refer to the Investigator’s Brochure for more detail on the safety and immunogenicity of ALVAC-HIV vaccines.

**4.0 EXPERIMENTAL DESIGN**

**4.1 Safety Evaluation**

Assessment of product safety will include clinical observation and monitoring of hematological, chemical, and immunologic parameters. Safety will be evaluated by monitoring participants for local and systemic adverse experiences during the course of the trial. Subjects will be closely observed for 30 minutes after immunization and then monitored in person by study staff 3 days post each immunization. Subjects will receive a diary card to record their temperature and measure local reaction to the injection. Patients will also be advised to maintain phone contact with study staff in the event of an adverse experience. Any phone contact that suggests a study related problem, an immediate in person evaluation will be scheduled. These efforts will focus on the toxicity parameters listed in Section 5.1. Also see Section 6.1 for power calculation for identifying adverse experiences.

**4.2 Immunogenicity Determinations**

The parameters that will be evaluated to determine vaccine immunogenicity include humoral and cellular immune responses to HIV-1. Assays for cellular immune response will primarily focus on CTL activity (see Protocol section 10). Assays for humoral immunity will include quantitation of both systemic and mucosal specific anti HIV antibodies. The selection of systemic and mucosal assays is the result of 3-4 years of developing expertise in the combined labs of Drs. Anton, Jamieson and Yang in optimizing sensitive detection assays able to be performed with limited numbers of cells acquired from mucosal biopsies.

**4.3 Detection of Intercurrent HIV-1 Infection**

See Section 10.3.1

**5.0 DATA AND SAFETY MONITORING**

- 1. **Risks**

The risks associated with delivery of the ALVAC-HIV (vCP205) in this trial are described in attached Investigators Brochure. The investigators of this project will continuously monitor data collected from the subjects in this project. Particular emphasis will be put on reviewing any adverse experiences that occur in the subjects. The following will be monitored:

1. Toxic reactions at the site of injection: pain, tenderness, erythema, induration, and other subject reported effects.
2. Systemic reactions: fever, muscle ache, headaches, nausea, fatigue, rashes, any hypersensitivity reactions
3. Immune responses: changes in CD4 and CD8 lymphocytes
4. Hematologic measures: changes in CBC, differential, platelets
5. Hepatic/renal functions: ALT, creatinine
6. Other neurologic, gastrointestinal or dermatologic changes
7. Behavioral changes: increases in sexual and other HIV-1 risk behavior
   1. **Adverse Experience Reporting Requirements**

Peter Anton, M.D. initially will review all adverse experience reports followed by the independent monitor. The independent monitor designated for the trial is Ronald Mitsuyasu M.D. Dr. Mitsuyasu is Director of the UCLA CARE Clinic for HIV/AIDS patients and has conducted numerous ACTG and other clinical trials at UCLA over the past 10 years. He was selected to monitor this trial because of both his clinical as well as clinical trial experience and because he has no financial or other interest in the outcome of the trial. To assure adequate safety monitoring Dr. Mitsuyasu (or his designated stand-in when he is out of area) and the Aventis Pasteur officer will be notified via e-mail of any Serious Adverse Experiences (SAE) as defined in the CFR 312.32 or grade 3 (see Severity Grading table attached) or higher adverse events and the Adverse Experiences Report (AER - attached) form will be completed and submitted within 48 hours. If the SAE or adverse events are grade 4 or 5, the above protocol will be followed with the addition of having the other agents (FDA and DAIDS project/medical officers informed). Dr. Anton will follow-up on each incident with Dr. Mitsuyasu and/or the FDA and DAIDS project/medical officers, as appropriate, to determine the subjects’ continued enrollment in the study. Dr. Mitsuyasu will have ultimate local authority to withdraw subjects from the study on the basis of safety concerns. All grade 1 and 2 events will be reported to all parties above at the end of the study. Because of the small number of subjects, intense schedule of individual monitoring and the paced enrollment of new subjects over the first year (due to the complexity of laboratory assays), Dr. Mitsuyasu will receive only individual data as the need arises, rather than aggregate data. Dr. Mitsuyasu will not be reviewing the immunologic data from this study. In addition, all reports will be forwarded to Aventis Pasteur.

1. **STATISTICAL ANALYSIS**

The results of the cellular immunology assays will be analyzed to determine the differences in immune responses at the baseline visit and each post-immunization visit. Comparisons of groups will be performed using either Fisher exact tests (categorical data) or exact Wilcoxon/Kruskal-Wallis tests (continuous data). Longitudinal analyses may be considered if appropriate, but due to small sample sizes these would be largely exploratory. We expect that the immunizations will stimulate significant cellular immune responses in humans and the levels of immune responses will increase as the number of inoculations with the vaccine increases. The relationship of the responses in the mucosa will also be expressed in relationship to those in the blood, e.g., 2-fold more than in the blood; 60% of the level in the blood, to facilitate comparisons between the immune responses in the groin and deltoid groups.

**Analysis:**

Interpretation to date from our previous vaccinia-based HIV vaccine trial is trending to support the belief that while peripheral immunization can induce peripheral immune responses, it does not necessarily induce mucosal responses. We have seen seroconversion peripherally with induced false-positive HIV ELISAs but effectively no indication of detected mucosal immune responses. This is in a setting of optimized assays and frequent samplings. Nevertheless, it is possible that sampling was scheduled at too great an interval following antigen exposure or the absorbed (humoral) or expanded (cellular) yields are too small to be reflective of mucosal responses. In the previous trial, we have demonstrated high trial adherence by subjects as well as optimization of mucosal assays (in press Journal Immunologic Methods). It is significant that using improved and optimized mucosal techniques; we are not finding mucosal responses in the presence of peripheral seroconversion.

The present protocol using vCP205, without boost, on a more condensed immunization schedule with more immediate mucosal assessments using the same immune response readouts will help elucidate whether these absences persist using a different vaccine with a different expression mechanism on a different schedule.

- 1. **Power calculations for identifying adverse experiences**

### Twelve subjects will receive the vaccine, 6 in the groin area, subcutaneously (SQ) and 6 in the deltoid region intramuscularly (IM). Six subjects will receive placebo, 3 in the groin SQ and 3 in the deltoid region IM. The table shows two sets of probabilities for observing adverse events. *Visit-specific* probabilities refer to adverse events that have a common, independent rate of occurrence for each of the four vaccination visits, i.e. the relevant n is the total number of injections. *Patient-specific* probabilities refer to adverse events that affect a given proportion of subjects, i.e. the relevant n is the number of patients. The first pair of columns give probabilities of observing one or more and two or more adverse events in a particular 6 patient arm. The next pair of columns gives probabilities for observing events in the 12 patient treatment group as a whole. The final pair of columns gives these probabilities for all 18 patients in the trial.

### If a given toxicity affects 5% of patients in the treatment groups, then the probability that one or more events are observed among the 12 subjects is 46% (91% if this event rate is visit-specific). If the toxicity occurs at this same 5% rate but in only one of the 6 patient arms (e.g. deltoid treatment), the corresponding probabilities of observing one or more events are 26% and 71%. There is 80% probability to detect one or more occurrences of an event with a patient-specific (visit-specific) rate of 24% (6.5%) with 6 subjects or a 13% (3.3%) event rate with 12 subjects (not shown in table).

| **Event Rate** | | **Probability of AE’s in a 6 patient arm of the trial** | | **Probablity of AE’s in 12 patient treatment group** | | **Probability of AE’s in entire patient group** | |
| --- | --- | --- | --- | --- | --- | --- | --- |
|  |  | **Pr{1 or more events }** | **Pr{2 or more events }** | **Pr{1 or more events }** | **Pr{2 or more events }** | **Pr{1 or more events }** | **Pr{2 or more events }** |
| Patient-specific adverse event rate | 1% | 0.059 | 0.001 | 0.114 | 0.006 | 0.165 | 0.014 |
|  | 5% | 0.265 | 0.033 | 0.460 | 0.118 | 0.603 | 0.226 |
|  | 10% | 0.469 | 0.114 | 0.718 | 0.341 | 0.850 | 0.550 |
|  | 20% | 0.738 | 0.345 | 0.931 | 0.725 | 0.982 | 0.901 |
|  | 30% | 0.882 | 0.580 | 0.986 | 0.915 | 0.998 | 0.986 |
|  | 40% | 0.953 | 0.767 | 0.998 | 0.980 | 0.999 | 0.999 |
|  | 50% | 0.984 | 0.891 | 0.999 | 0.997 | 0.999 | 0.999 |
| Visit-specific adverse event rate | 1% | 0.214 | 0.024 | 0.383 | 0.083 | 0.515 | 0.162 |
|  | 5% | 0.708 | 0.339 | 0.915 | 0.699 | 0.975 | 0.881 |
|  | 10% | 0.920 | 0.708 | 0.994 | 0.960 | 0.999 | 0.995 |
|  | 20% | 0.995 | 0.967 | 1.000 | 1.000 | 1.000 | 1.000 |
|  | 30% | 1.000 | 0.998 | 1.000 | 1.000 | 1.000 | 1.000 |
|  | 40% | 1.000 | 1.000 | 1.000 | 1.000 | 1.000 | 1.000 |
|  | 50% | 1.000 | 1.000 | 1.000 | 1.000 | 1.000 | 1.000 |

**7.0 RANDOMIZATION AND BLINDING**

This is a randomized, modified double-blind placebo-controlled study. The 18 subjects in the study will be randomized first into either the deltoid or groin group, then to the treatment or placebo arm. The randomization plan is based on three groups of six patients each so that within each sequential cohort of 6 recruited subjects, three will randomly be assigned to groin and the other three will receive deltoid immunization. Each of these subgroups will also be randomized so that two subjects will receive active treatment whereas the other will receive placebo. This approach was felt necessary to achieve a balanced design, even if the study does not recruit all planned subjects.

The vaccine and placebo have a slightly different appearance. In order to maintain the modified blind a trained RN or Nurse Practitioner will deliver the vaccine or placebo not otherwise associated with the study. The study staff will handle all processes relating to subject study participation, care and monitoring except for actual vaccination. Staff will remain in the immediate vicinity of the subject during vaccination.

- 1. **HUMAN SUBJECTS**

Each subject must be a human immunodeficiency virus type 1 (HIV-1) seronegative man or non-pregnant woman with a low risk profile for HIV-1, and at least 18 years old.

- 1. **Inclusion Criteria**

1. Age: 18 – 60 years of age.
2. Female or male (for females, negative serum pregnancy test at time of entry into the study and assurance of adequate, reliable birth control measures until at least 60 days after the final immunization or documentation of non-reproductive status e.g. record of tubal ligation).
3. At low risk for acquiring HIV-1 (no STD within one year of beginning trial, no history of injection drug use, no sex with an HIV infected individual or active injection drug user within six months of beginning trial, no unsafe sexual activity with unknown partners or mutually monogamous relationship with a known HIV seronegative partner (per subject report) for the last six months.
4. Normal history and physical examination.
5. Normal complete blood count, differential, ALT and creatnine.
6. Negative for HIV by an FDA approved blood test, negative hepatitis B surface antigen and anti-HCV or negative HCV PCR if anti-HCV positive.
7. Capable of giving informed consent.
8. Willing to abstain from receptive anal intercourse during the 14 months of the study
9. Available for follow-up during the 14 month duration of the study.
   1. **Exclusion Criteria**
10. Pregnant or lactating women.
11. Allergy to eggs or neomycin
12. Vaccination with live attenuated vaccines within 60 days of study [medically indicated subunit or killed vaccines (e.g., influenza, pneumococcal) are not exclusionary, but should be given at least one month prior to first immunization, and not until 14 days following the last immunization.
13. Significant gastrointestinal complaints such as inflammatory bowel disease or chronic diarrhea.
14. Immunosuppression of any type including those related to lupus, rheumatoid arthritis, leukemia, lymphoma, generalized malignancy, agammaglobulinemia, therapy with alkylating agents, antimetabolites, or radiation.
15. Recent (past 6 months) or current use of immunosuppressive medications; i.e. oral, parenteral and orally inhaled corticosteroids, and/or cytotoxic medications.
16. Thyroid disease including history of thyroidectomy and diagnoses requiring medication
17. Unstable asthma, exposure to or active tuberculosis, seizure disorders, bleeding disorders, splenectomy, hypertension (less than 150/100 if on medication).
18. Medical or psychiatric condition or occupational responsibilities that preclude subject compliance with the protocol. Specifically excluded are persons with a history of suicide attempts, recent suicidal ideation or who have past or present psychosis.
19. Prior receipt of HIV-1 vaccines or placebo in previous HIV vaccine trial
20. Receipt of blood products 120 days prior to HIV screening
21. Receipt of immunoglobulins 60 days prior to HIV screening
22. Any history of anaphylaxis or history of other serious adverse reactions to vaccines.
23. History of serious allergic reaction to any substance requiring hospitalization or emergent medical care (e.g., Stevens-Johnson syndrome, bronchospasm, or hypotension).
24. History of non-prescribed injection drug use.
25. Use of experimental agents within 30 days prior to study.
    1. **Benefit of Participation**

This study is not thought to be of direct benefit to the subject. The possible benefit to humanity and the scientific community includes a better understanding of the human immune system and resistance to HIV infection, which may lead to improved treatments and/or vaccines against HIV infection.

To defray expenses and compensate participants for their time, they will be paid for participation in this study. Each participant will be paid $25 for each blood donation or clinical exam visit, $200 for immunization visits which may include a blood donation and $100 for the flexible sigmoidoscopy with mucosal biopsy visits which may include a blood donation. Participants will be paid in cash at each visit. All subjects who have positive HIV-1 serology at the end of the study as measured by an FDA-approved HIV-1, 2 ELISA and/or Western Blot kit, will be offered follow-up HIV-1 diagnostic testing by HIV-1 RNA PCR through the Anton Lab after the completion of the study. In addition, the participants will be given $750 to cover the approximate clinical cost of three PCR tests when the participant or his/her healthcare provider feels it is indicated. This is based on the current average cost of $250 per test. Participants will also be provided with transportation, if needed, or parking at the clinical site on the day of any procedure.

**8.4 Withdrawal from study**

**8.4.1 Subject-initiated withdrawal**

If any participant should choose to withdraw from the study, they will be informed through the consent that it will not affect their relationship with UCLA Medical Center, or their right to health care or other services to which they are otherwise entitled. Additionally, if a volunteer decides not to participate, he or she is free to withdraw consent and discontinue participation at any time without prejudice to future care at UCLA.

**8.4.2 Investigator-initiated withdrawal**

The investigator may withdraw a subject from participating in this research if circumstances arise which warrant doing so. The investigator, Dr. Peter Anton, will make the decision and inform participants of these circumstances. This decision may be made either to protect participants’ health and safety, because a condition develops that places the participant in an exclusion category or because baseline responses will not permit an effective comparison with post-immunization results.

Experiences of grade 4 (potentially life threatening) will automatically exclude subjects from further participation in the trial. Dr. Anton and Dr. Mitsuyasu will evaluate all SAEs and grade three (severe) experiences to determine if the episode represents an ongoing risk related to the study and make a determination about the subject’s further participation in the study. Grade 1 and 2 experiences will generally not be exclusionary unless they are related to the immunization and progressive in degree or persistent.

The decision to withdraw a participant may be made in the case of:

- Grade 4 adverse experiences
- Grade 3 adverse experiences related to the immunization and a determination that the duration or other factor indicates withdrawal is in the best interest of the subject
- Serious Adverse Experiences regardless of grade of individual event
- Grade 1 and 2 experiences related to the immunizations that are progressive in degree or persistent.
- HIV-1 infection
- Pregnancy

**8.4.3 Withdrawal Due to HIV-1 Infection**

If tests indicate that the subject is infected with HIV, either the subject or their primary health care provider will be notified (depending upon which method the subject has selected on the consent form). If the subject will be informed directly, a research staff member who is trained and experienced in counseling individuals in this area will work with the subject. The subjects will be offered referrals to assist with any emotional, medical, or other support needs they may have relating to HIV infection.

**9.0 IMMUNIZATION PROCEDURES**

- 1. **ALVAC-HIV (vCP205)**
     1. **Preparation of Dosing Solution:**

Aventis Pasteur ALVAC-HIV (vCP205) lot # S3673 at a 1x 10^6.87^ TCID_50_ will be used. Prior to injection, each dose of vaccine is reconstituted by dissolution of the lyophilisate with the diluent supplied, i.e., sterile saline solution NaCl 0.4%. The diluent will be injected slowly into the vial containing the lyophilized ALVAC. Then, the vial will be swirled gently until dissolution of the lyophilisate. Avoid inverting the vial. The reconstituted vaccine must be kept at 2-8°C and used within two hours. Do not freeze.

**ALVAC Placebo** is supplied as a sterile, lyophilized product that consists of a mixture of virus stabilizer, and freeze drying medium. The diluent supplied for reconstitution of ALVAC-Placebo consists of sterile 0.4% NaCl and conforms to established requirements for sterility, safety, and pyrogen testing. The volume of the prepared vaccine is 1 mL.

- - 1. **Administration of ALVAC-HIV (vCP205)**

The vaccine will be prepared by the UCLA Research Pharmacy within two hours of administration to the subject. The vaccine or placebo is preassigned to the subject’s study ID number. The preparing pharmacist and a second pharmacist will note the subject study ID number, dose and vaccine or placebo in the dispensation log and on the label of the syringe. In order to maintain blinding, the vaccination will be administered by a trained Registered Nurse, Nurse Practitioner, Physician or similar licensed staff not otherwise involved with the safety and monitoring of the subjects. The person administering the vaccine or placebo will also document the date and drug randomization, as noted on the label of the syringe, in a separate log that will be kept in the General Clinical Research Center.

Immunization. All four immunizations with ALVAC-HIV (vCP205) will utilize the same dilution.

Clean the skin with alcohol

- Draw 1 ml of the vaccine into a syringe for standard subcutaneous (groin) or intramuscular (deltoid) injection.
- Using a 25g 5/8” needle, administer the injections subcutaneously into the groin area avoiding femoral arteries and nerve pathways. Groin injections will be placed unilaterally, medial to the femoral artery, vein and nerve (approximately halfway between the anterior superior iliac spine and the symphysis pubis) and anterior to the horizontal group of superficial inguinal lymph nodes. Immunization site will be unilateral groin delivery. Immunizations #1 and #3 will be administered to the subjects’ right side and immunizations #2 and #4 will be administered to their left.

-or-

- Using a 21g 1” needle, administer the injections intramuscularly into the deltoid region. Deltoid injections will be placed in the midlateral aspect of the deltoid muscle, 5-8 cm inferior to the scapular acromion process and the lateral edge of the clavicle. Immunization site will be unilateral deltoid delivery. Immunizations #1 and #3 will be administered to the subjects’ right side and immunizations #2 and #4 will be administered to their left.
  - 1. **Route of Immunization**

The vaccine or placebo will be administered subcutaneously (1 ml) unilaterally to inguinal region (alternating sides each sequential immunization), medial to the femoral artery, vein and nerve or IM (1 ml) unilaterally to deltoid region (alternating sides each sequential immunization)

- 1. Vaccine Administration Schedule

| Route/Volume of ALVAC-HIV (vCP205) or placebo | IMMUNIZATIONS | | | |
| --- | --- | --- | --- | --- |
|  | 1 (day 0) | 2 (day 7) | 3 (day 14) | 4(day 21) |
| Subcutaneous  or  Intramuscular  1 ml | ALVAC-HIV (vCP205) | ALVAC-HIV (vCP205) | ALVAC-HIV (vCP205) | ALVAC-HIV (vCP205) |
|  | placebo | placebo | placebo | placebo |

- 1. **Disposal**

Partially-used and empty vials will be destroyed on site after final vaccine accountability is performed by crushing or incineration according to UCLA Environmental Health and Safety biohazard procedures. All expired or unused vaccine vials will be returned to Aventis Pasteur S.A. Study supplies, including partially used vials, will not be administered to other subjects or used for *in vitro* or animal experiments. All used syringes and needles will be placed into a biohazard “sharps” container and disposed of following the designated UCLA procedures for disposal of potentially infectious materials. Disposal of all materials will be done in accordance with UCLA “Safety Procedures-Standard Operating Procedures”.

1. **IMMUNOLOGIC AND VIROLOGIC STUDIES TO BE PERFORMED**
   1. **Safety**

Assessment of product safety will include extensive monitoring of physical, hematologic, chemistry and immunologic parameters, as well as subjects’ self-report of symptoms/responses.

- 1. **Plan for Evaluation of Immune Responses**

**10.2.1 Overview of Procedures and Immunologic Assays**

Immunizations 1, 2, 3 and 4 will be given at days 0,7,14 and 21, respectively. Mucosal biopsies and rectal secretions will be obtained at days –28 and –14 (baselines) and at days 10 and 24 and 6 and 12 months post day 0 (to assess durability of response). Mucosal biopsy specimens (up to 20 per donor /per procedure) will be obtained and processed within 15 minutes of harvest of the biopsies. We will also collect and test plasma for antibody days -42, -14, 10, 17, 24 and 6 and 12 months. Blood will be obtained at days -42, -28, -14, 0, 7, 10, 14, 17, 24, months 6 and 12 (and as needed per subject request for HIV status) to characterize the cellular immune responses and quantitative lg responses.

10.2.2 Measurement of HIV-1-specific antibodies in rectal and oral secretions

At both baseline and each post-immunization sigmoidoscopy visit we will measure HIV-1 antibodies from rectal secretions using the Weck-Cel method and from oral secretions using OraSure®. This involves collection of fluids into Weck-Cel sponges per a protocol developed by Kozlowski et.al. (JAIDS 24:297-309, 2000) or onto the OraSure® plegette according to the package insert. The pre-moistened sponges are inserted 5cm into the rectum of the subject via lubricated applicator pipette. The sponge is held in for 5 minutes and withdrawn. The procedure is repeated with a second sponge at the same location. The plegettes are inserted into the mouth, rubbed along the buccal mucosa few times and held there for 2 minutes. The absorbed antibodies are eluted by centrifugation in a two step process with elution buffer containing protease inhibitors into a pre-weighed collection tube. The collected fluids are diluted 1/10 in ELISA diluent and assayed for HIV–specific antibodies using the Vironostika® HIV-1 ELISA kit as per the manufacturer’s instructions with the inclusion of a standard curve generated from a purified anti-p-24 antibody (ImmunoDiagnostics, Inc). In addition, samples are assayed for total IgG, IgM and IGA content by ELISA. In brief, samples are incubated in wells pre-coated with anti-human IgG, IgM or IgA (DAKO Corp) washed and developed using horse-radish peroxidase conjugated secondary antibodies (Dako Corp) and OPD substrate. Purified human immunoglobulin standards (Jackson Labs) are included for each immunoglobulin and standard curves are generated. The total immunoglobulin content of the samples is calculated by the sum of the IgG, IgM and IgA. Results are expressed as units of HIV antibody per 100ug of total immunoglobulin. The collection and quantification assays for assessing secreted mucosal antibodies (rectal and oral) have been validated.

**10.2.3 Expansion of mucosal lymphocytes in vitro**

Using bi-specific antibodies provided by Dr. Johnson Wong, isolated mucosal lymphocytes are expanded in a polyclonal manner through co-stimulation of CD3 and CD4 and CD3 and CD8. Using this protocol, 1 X 10^5^ lymphocytes can be expanded into several million CD4^+^ or CD8^+^ lymphocytes. Because the expansion is driven by antibodies to surface antigens and not by viral antigens, this method expands T-cells in a polyclonal manner and therefore should not significantly alter relative frequencies of T-cell subpopulations. Expanded cells both PBMC and MMC will be used in chromium release assays, ELISPOT, and proliferative assays. For this assay, 10^5^ gut lymphocytes, or 10^5^ freshly ficolled PBMC are set up in a well of a 24-well plate containing: 10^5^ lymphocytes to be expanded + 10^6^ irradiated (3000 rad) autologous PBMC in 2ml of RPMI with 10%FCS and 50U/ml IL-2 (R10-50) containing 2μg/ml of a bispecific CD3:CD4 antibody or the bispecific CD3:CD8 antibody.

**10.2.4 ELISPOT Analysis for IFN-γ for fresh PBMC, expanded PBMC and MMC**

Enzyme Linked-ImmunoSpot (ELISPOT) assay is performed as described by Tary-Lehmann et al. Briefly, 1 X 10^5^ to 3 X 10^5^ lymphocytes are plated in 96 well Unifilter plates (Whatman Inc. Cliffton, NJ) that have been incubated with INF-γ antibodies (4 μg/ml) (Pharmingen, Carlsbad, CA) and blocked with PBS-BSA. For antigen stimulation: 100 μl of canary pox virus (either ALVAC or ALVAC-HIV (vCP205) and HIV specific peptides are added to the plate at 5 pfu/cell. As a positive control, antibodies to CD2/2R and CD28 (Becton Dickinson Immunocytometry Systems, San Jose, CA) are added at 5 μg/ml and 20 μg/ml respectively. After 24 hours at 37^o^C, the plate is washed, and 100 μl biotinylated IFN-γ (2μg/ml) (Pharmingen, Carlsbad, CA) added and incubated for 24 hours at 4^o^C. After washing, 100 μl diluted (1:2000) Streptavidin-HRP (Pharmingen, Carlsbad, CA) is added for 2 hours. The plate is washed again and the color developed by the addition of 200 μl of visualization solution (24 ml of 0.1M acetate buffer with 800 μl of AEC solution [10mg AEC/ml DMF] and 12 μl of H_2_O_2_ [30%]) and subsequent incubation for 20 minutes at room temperature in the dark. Tap water is used to stop the reaction and the plate is air-dried. An ImmunoSpot Analyzer will be used for computerized analysis.

- - 1. **Chromium release assay for cytotoxicity**

Chromium release assays will be performed on the expanded CD8^+^ cell lines (See 10.2.3 above) as described in the preliminary results per standard protocol. Briefly, autologous EBV-transformed B-cell lines will be infected overnight with recombinant vaccinia virus for use as target cells. These cells will be labeled with ^51^Cr. In a 96-well U-bottom plate, 10^4^ target cells will then be plated with effector-target cell ratios of 12.5:1, 25:1, 50:1 in duplicate for a 4 hour incubation, followed by determination of supernatant ^51^Cr on 96 well scintillation plates (LumaPlate, Packard; MicroBeta 1450, Wallac).

**10.2.6 CD4 T cell proliferation measured by ELISPOT Analysis for IL-2**

CD4 T cells will be assayed either immediately following isolation or after non-specific expansion as described above. Briefly 1 x 10^5^ to 3 X 10^5^ CD4 cells will be plated in 96 well Unifilter plates that have been coated with IL-2 antibodies and blocked with PBS-BSA. For antigen stimulation: 100ul of canary pox virus (either ALVAC or ALVAC-HIV (vCP205) at 5pfu/cell or HIV specific peptides at 100 ug/ml. As a positive control, antibodies to CD2/2R and CD28 are added at 5 and 20ug/ml respectively. After 24 hours at 37°C the plate is washed and 100ul of biotinylated IL-2 (2ug/ml) is added and incubated for 24 hours at 4°C. After washing, 100ul of diluted (1:2000) streptavidin-HRP is added for 2 hours. The plate is washed again and the color developed by the addition of 200 ul of visualization solution and subsequent incubation for 20 minutes at room temperature in the dark. Tap water is used to stop the reaction and the plate is air-dried. An ImmunoSpot Analyzer will be used for computerized analysis. The assays have been performed by the laboratory using PBMC with sensitive reproducible and specific results.

## 10.2.7 ELISA based assay for RANTES production

## To culture lymphocytes (PBMC or MMC) for the detection of *in vitro* RANTES production, 4 x 10^4^ to 4 x 10^6^ cells are cultured in IL-2 (1000 units/ml) in 24 well, flat bottom plates (Becton Dickinson, San Jose, CA). For non-specific stimulation, PWM (10 ug/ml), PHA (5 ug/ml), or antibodies to CD2/2R and CD28 (Becton Dickinson, San Jose, CA) are added at 10 μg/ml and 20 μg/ml respectively. Antigenic stimulation will be performed as described above for ELISPOT. 1 ml supernatants are harvested at 24 hr, day 10, and day 14 and 1 ml of fresh media added. For quantitation, samples are diluted 1:2 in RPMI with 10% FCS and plated with 100 μl of assay diluent (RD1A) on the RANTES ELISA plate (R&D Systems, Minneapolis, MN). When needed, supernatants have been further diluted 4-, 6-, or 10-fold. 100 μl of standard provided in the ELISA Kit (R&D Systems, Minneapolis, MN) is added to the plate as well and incubated at room temperature for 2 hours. The plates are washed, conjugate is added at 200 μl per well and the plate is incubated at room temperature for 1 hour. Plates are washed and 200 ml of substrate solution is added and the plates are incubated for 20 minutes at room temperature. 50 ml of stop solution is added and the plate is read at 450 nm on an ELISA reader (Spectra Max 340 from Molecular Devices, Sunnyvale, CA).

**10.2.8 Quantitative image analysis (QIA) for *in vivo* RANTES production**

Cryopreserved colon biopsies, embedded in OTC-compound (Tissue-TEK, Mites, Elkhart, IN), will be cut in 8 µm thick sections and fixed with 2% formaldehyde. Slides will be incubated with polyclonal affinity-purified, biotinylated RANTES (R&D Systems, Minneapolis, MN), followed by an avidin-biotin horseradish peroxidase complex (Vectastain elite kit, Vector Laboratories, Burlingame, CA, USA) and counterstained with hematoxylin. Digital images of stained samples will then be transferred from a DMR-X microscope (Leica, Wetzlar, Germany), into a computerized image analysis system, Quantimet 550IW, (Leica, Cambridge, UK), which allows detection of 16.7 million different colors. For analysis, percentage positive area in the total of 2-8 fields, depending on the size of the biopsy, with a total mean area of 2.2 X 10^5^ μm^2^, will be assessed for positive-stained area and the total area of hematoxylin positive cells present. Semiquantitative results will be expressed as percentage positive area of total tissue area.

**10.2.9 Cytotoxic T lymphocyte epitope mapping**

We will non-specifically expand CD8^+^ cells from the gut and peripheral blood. As also shown in the Preliminary Results, a further round of antigen specific stimulation will be performed, using recombinant vaccinia virus-infected autologous EBV-transformed B-cells, as per the method of Lubaki et al. This will be followed by limiting dilution cloning and fine mapping as per published methods. We have already successfully isolated HIV-1-reactive clones from the peripheral blood of an infected individual using these methods.

**10.2.10 Flow cytometric analysis for α4β7 expression on PBMC and activation markers (HLA-DR and CD-38) on PBMC and MMC**

Expression of α4β7 is required for successful homing of peripheral lymphocytes to the gastrointestinal compartment. As an indicator of the percentage of blood lymphocytes which can potentially home to GALT in response to vaccination, immunofluorescent staining will be performed on PBMC isolated from whole blood by Ficoll-Hypaque. One million PBMC will be co-stained with antibodies against CD3, CD4, CD8 (Becton Dickinson, San Jose, CA) and α4β7 (Integrin β7: BD Pharmingen, San Diego CA). Cells will also be stained in parallel with isotype controls. Samples will be assessed on a FACSCalibur flow cytometer. Similar protocols will be performed assessing changes in HLA-DR and CD-38 (Becton Dickinson, San Jose, CA) expression on CD4 and CD8 T lymphocyte subsets in both freshly acquired PMBC and MMC.

- 1. **HIV-1 Virology**
     1. **Detection of intercurrent HIV-1 infection by HIV-1 PCR**

The ALVAC-HIV (vCP205) vaccine includes *gag* and *pol* gene products as well as *env* (gpl60), in its composition. Consequently, the confirmatory Western Blot serologic assay may be unable to distinguish a vaccine-induced immune response from natural infection in study subjects. Overall, several precautionary measures will be taken to clarify this distinction between vaccine-induced antibody responses and those from intercurrent HIV-1 infection:

1. Only volunteers at lower risk for HIV-1-infection will be considered for enrollment. Volunteers will be counseled frequently during the trial on avoidance of HIV-1 infection.

2. Subjects will have frequent clinical evaluations for signs or symptoms of an acute HIV-1 infection syndrome. An intercurrent illness consistent with HIV-1 infection or circumstances suggesting increased risk of acquiring HIV-1 (e.g., report of higher risk behavior, newly acquired STD, report of unsafe sexual activity with an HIV-1-infected individual) would prompt a diagnostic work up.

1. Periodic HIV-1 ELISAs and Western Blots will be performed. If intercurrent HIV-1 infection is suspected, further diagnostic work-up will be performed.

All subjects who have positive HIV-1 serology at the end of the study as measured by an FDA-approved HIV-1, 2 ELISA and/or Western Blot kit, will be offered follow-up HIV-1 diagnostic testing by HIV-1 RNA PCR through the Anton Lab after the completion of the study. In addition, the participants will be given money to cover the approximate clinical cost of three PCR tests when the participant or his/her healthcare provider feels it is indicated.

- 1. **Calendar of Studies**

The subjects will have 3 baseline visits with blood draws and two sigmoidoscopies to establish baseline measurements of immune function and screen for any exclusion criteria. They will then begin an intensive series of vaccinations and measurements for safety and immunologic response. Subjects will have a total of 880 ml of blood drawn over the 14 months of the study. No more than 450 ml will be drawn in any 8 week period. The subjects will be encouraged to report any unusual symptoms that may be related to the study and may make a visit with clinical staff at any point between any of the following study visits. The scheduled intervals between visits may vary by several days due to scheduling or other difficulties.

## Day - 42(Screening)

- Signed enrollment consent form
- Completion of Participant Profile form, including medical history
- Counseling on avoidance of HIV-1 infection and pregnancy – handout signed
- Complete clinical evaluation (temperature, vital signs, and physical exam)
- Complete STD examination
- Blood draw as follows
- 10 ml untreated “SST” - chem panel & HCG
- 35 ml untreated “Red Top” for Hepatitis B and C antibody screening (10ml), HIV-1 antibody ELISA (10ml) canarypox antibody – research (10 ml) RPR (5ml)
- 20 ml EDTA “Lavender Top” - for CBC (5ml), CD4/8 & HLA phenotyping (5ml), HIV-1 RNA PCR (10ml)
- 60 ml heparinized “Green Top” – for cryopreservation of feeder cells

# Day -28 (Baseline immune responses; mucosal biopsies #1)

- Counseling on avoidance of HIV-1 infection and pregnancy – handout signed
- Blood draw as follows:
- 10 ml EDTA “Lavender Top” for HIV-1 antibody - research
- 90 ml heparinized “Green Top” for CTL assays
- Weck-Cel and OraSure secretion collection
- Sigmoidoscopy (up to 20 specimens collected for baseline screening for CTL activity)
- Give participant post-procedure home care instructions

# Day -14 (Baseline immune responses; mucosal biopsies #2)

- Counseling on avoidance of HIV-1 infection and pregnancy – handout signed
- Blood draw as follows:
- 10 ml untreated “Red Top” for HIV-1 antibody
- 15 ml EDTA “Lavender Top” for HIV-1 antibody – research (10ml) HIV RNA PCR (5ml)
- Weck-Cel and OraSure secretion collection
- Sigmoidoscopy (up to 20 specimens collected for baseline screening for CTL activity)
- Give participant post-procedure home care instructions

### Day 0 (Immunization #1)

- Limited clinical evaluation
- Urine test for pregnancy if female w/ child bearing potential
- Risk reduction counseling on avoidance of HIV-1 infection and pregnancy-handout signed
- Blood draw as follows:
- 30 ml heparinized “Green Top” – for CTL assays
- Immunization administered subcutaneously to the left or right groin area or IM in the left or right deltoid area
- Participant will be observed for 30 minutes
- Give participant post immunization counseling and home care instructions

**Day 3 (approximately 3 days post immunization; safety)**

- AE assessment
- Note changes in injection site and surrounding area

##### Day 7 (Immunization #2; safety and immune responses)

##### AE assessment

##### Risk reduction counseling on avoidance of HIV-1 infection and pregnancy Examination of site of immunization

- Note changes in injection site and surrounding area
- Blood draw as follows:
- 5 ml untreated “SST ” for chem panel
- 15 ml EDTA “Lavender Top” for CBC (5ml), CD4/8 count (5ml), HIV RNA PCR (5ml)
- Immunization administered subcutaneously to the left or right groin area or IM in the left or right deltoid area on the side alternate to previous injection
- Give participant post immunization counseling and home care instructions

**Day 10 (approximately 3 days post immunization; safety** **and immune responses; mucosal biopsies # 3)**

- AE assessment
- Examination of sites of immunization
- Note changes in injection site and surrounding area
- Blood draw as follows:
- 10 ml untreated “Red Top” for HIV-1 antibody
- 10 ml EDTA “Lavender Top” for HIV-1 antibody - research
- 60 ml heparinized “Green Top” for CTL assays
- Weck-Cel and OraSure secretion collection
- Sigmoidoscopy (up to 20 specimens collected)

##### Day 14 (Immunization #3; safety and immune responses)

##### AE assessment

##### Urine test for pregnancy

##### Risk reduction counseling on avoidance of HIV-1 infection and pregnancy

- Blood draw as follows
- 5 ml untreated “SST” for chem panel
- 15 ml EDTA “Lavender Top” for CBC (5ml), CD4/8 count (5ml) HIV RNA PCR (5ml)
- Immunization administered subcutaneously to the left or right groin area or IM in the left or right deltoid area on the side alternate to previous injection
- Participant will be observed for 30 minutes
- Give participant post immunization counseling and home care instructions

**Day 17 (3 days post-immunization #3; safety and immune responses)**

- AE assessment
- Risk reduction counseling on avoidance of HIV-1 infection and pregnancy-handout signed
- Examination of sites of immunization
- Note changes in injection site and surrounding area
- Blood draw as follows:
- 10 ml untreated “Red Top” for HIV-1 antibody ELISA (10ml)
- 10 ml EDTA “Lavender Top” for HIV-1 antibody - research
- 60 ml heparinized “Green Top” for CTL assays

**Day 21 (Immunization #4; safety and immune responses)**

- AE assessment
- Risk reduction counseling on avoidance of HIV-1 infection and pregnancy-handout signed
- Examination of sites of immunization
- Note changes in injection site and surrounding area
- Immunization administered subcutaneously to the left or right groin area or IM in the left or right deltoid area on the side alternate to previous injection
- Participant will be observed for 30 minutes
- Give participant post immunization counseling and home care instructions

## Day 24 (3 days post-immunization #4; safety and immune responses; mucosal biopsy #4)

## AE assessment

- Risk reduction counseling on avoidance of HIV-1 infection and pregnancy
- Examination of sites of immunization
- Note changes in injection site and surrounding area
- Blood draw as follows:
- 5 ml untreated “SST” for chem panel
- 20 ml untreated “Red Top” for HIV-1 antibody ELISA (10ml) canarypox antibody – research (10ml)
- 25 ml EDTA “Lavender Top” for CBC (5ml), CD4/8 count (5ml) HIV RNA PCR (5ml) HIV antibody - research (10ml)
- 60 ml heparinized “Green Top” for CTL assays
- Weck-Cel and OraSure secretion collection
- Sigmoidoscopy (up to 20 specimens collected)
- Give participant post-procedure home care instructions

###### Month 6 (5 months post-immunization #4; safety and immune responses; mucosal biopsies #5)

## AE assessment since last visit

- Risk reduction counseling on avoidance of HIV-1 infection and pregnancy
- Examination of sites of immunization
- Note changes in injection site and surrounding area
- Blood draw as follows:
- 5 ml untreated “SST” for chem panel
- 20 ml untreated “Red Top” for HIV-1 antibody ELISA (10ml) canarypox antibody research (10 ml)
- 25 ml EDTA “Lavender Top” for CBC (5ml), CD4/8 count (5ml) HIV RNA PCR (5ml) HIV antibody - research (10ml)
- 90 ml heparinized “Green Top” for CTL assays
- Weck-Cel and OraSure secretion collection
- Sigmoidoscopy (up to 20 specimens collected)
- Give participant post-procedure home care instructions

###### Month 12 (11 months post-immunization #4; immune responses, mucosal biopsies #6)

## Limited clinical evaluation

## Risk reduction counseling on avoidance of HIV-1 infection and pregnancy

- Blood draw as follows:
- 5 ml untreated “SST” for chem panel
- 10 ml untreated “Red Top” for HIV-1 antibody ELISA
- 25 ml EDTA “Lavender Top” for CBC (5ml), CD4/8 count (5ml) HIV RNA PCR (5ml) HIV antibody – research (10ml)
- 90 ml heparinized “Green Top” for CTL assays
- Weck-Cel and OraSure secretion collection
- Sigmoidoscopy (up to 20 specimens collected)

###### As needed

###### Risk reduction counseling on avoidance of HIV-1 infection

- Blood draw as follows:
- 10 ml EDTA “Lavender Top” for HIV-1 RNA PCR
  1. **Summary of Studies**

| DAY | -42 | -28 | -14 | 0 | 3 | 7 | 10 | 14 | 17 | 21 | 24 | Mo. 6 | Mo. 12 |  |
| --- | --- | --- | --- | --- | --- | --- | --- | --- | --- | --- | --- | --- | --- | --- |
| VISIT | 1 | 2 | 3 | 4 | 5 | 6 | 7 | 8 | 9 | 10 | 11 | 12 | 13 | PRN |
| Immunization |  |  |  | X |  | X |  | X |  | X |  |  |  |  |
| Sigmoidoscopy, Weck & OraSure |  | X | X |  |  |  | X |  |  |  | X | X | X |  |
| AE assessment |  | X | X | X | X | X | X | X | X | X | X | X | X |  |
| Risk Reduction Counseling |  | X | X | X | X | X | X | X | X | X | X | X | X | X |
| STD Examination | X |  |  |  |  |  |  |  |  |  |  |  |  |  |
| Injection Site Evaluation |  |  |  |  | X | X | X | X | X | X | X |  |  |  |
| **PBMC’s** |  |  |  |  |  |  |  |  |  |  |  |  |  |  |
| CBC, differential, platelets | X |  |  |  |  | X |  | X |  |  | X | X | X |  |
| T-Cell count | X |  |  |  |  | X |  | X |  |  | X | X | X |  |
| CD38* | X |  |  |  |  | X |  | X |  |  | X | X | X |  |
| α4β7* | X |  |  |  |  | X |  | X |  |  | X | X | X |  |
| B-cell line established** | X |  |  |  |  |  |  |  |  |  |  |  |  |  |
| HLA typing** | X |  |  |  |  |  |  |  |  |  |  |  |  |  |
| ^51^CR |  | X |  | X |  |  | X |  | X |  | X | X | X |  |
| ELISPOT*** |  | X |  | X |  |  | X |  | X |  | X | X | X |  |
| Cryo PBMC feeder | X |  |  |  |  |  |  |  |  |  |  |  |  |  |
| **Plasma** |  |  |  |  |  |  |  |  |  |  |  |  |  |  |
| HIV-1 RNA PCR | X |  | X |  |  | X |  | X |  |  | X | X | X | X |
| HIV Ab Research IgG, IgA |  | X | X |  |  |  | X |  | X |  | X | X | X |  |
| **Serum** |  |  |  |  |  |  |  |  |  |  |  |  |  |  |
| Chem panel | X |  |  |  |  | X |  | X |  |  | X | X | X |  |
| Pregnancy test (HCG) | X |  |  |  |  |  |  |  |  |  |  |  |  |  |
| HIV-1 antibody ELISA | X |  | X |  |  |  | X |  | X |  | X | X | X |  |
| HIV Ab Res - canarypox | X |  |  |  |  |  |  |  |  |  | X | X |  |  |
| RPR | X |  |  |  |  |  |  |  |  |  |  |  |  |  |
| HBV/HCV Ab | X |  |  |  |  |  |  |  |  |  |  |  |  |  |
| **Urine** |  |  |  |  |  |  |  |  |  |  |  |  |  |  |
| Pregnancy test |  |  |  | X |  | X |  | X |  | X |  |  |  |  |
| **Tissue** |  |  |  |  |  |  |  |  |  |  |  |  |  |  |
| QIA on frozen spec |  | X | X |  |  |  | X |  |  |  | X | X | X |  |
| **MMC’s** |  |  |  |  |  |  |  |  |  |  |  |  |  |  |
| TruCount |  | X | X |  |  |  | X |  |  |  | X | X | X |  |
| DR38 |  | X | X |  |  |  | X |  |  |  | X | X | X |  |
| Expansion |  | X | X |  |  |  | X |  |  |  | X | X | X |  |
| **Expanded PBMC** |  |  |  |  |  |  |  |  |  |  |  |  |  |  |
| ELISPOT*** |  | X |  | X |  |  | X |  | X |  | X | X | X |  |
| Proliferative assay |  | X |  | X |  |  | X |  | X |  | X | X | X |  |
| **Expanded MMC** |  |  |  |  |  |  |  |  |  |  |  |  |  |  |
| ELISPOT*** |  | X | X |  |  |  | X |  |  |  | X | X | X |  |
| Proliferative assay |  | X | X |  |  |  | X |  |  |  | X | X | X |  |
| **Cultured PBMC** |  |  |  |  |  |  |  |  |  |  |  |  |  |  |
| RANTES |  | X |  | X |  |  | X |  | X |  | X | X | X |  |
| IgA, IgG |  | X |  | X |  |  | X |  | X |  | X | X | X |  |
| **Cultured MMC** |  |  |  |  |  |  |  |  |  |  |  |  |  |  |
| RANTES |  | X | X |  |  |  | X |  |  |  | X | X | X |  |
| IgA, IgG |  | X | X |  |  |  | X |  |  |  | X | X | X |  |
| * included in T cell count panel ** taken from other tubes *** includes IFN g, IL-2 and mapping | | | | | | | | | | | | | | |

**11.0 REFERENCES**

1. World Health Organization. Weekly Epidemiological Record. 49, 417-424. 12-6-2002. Report

2. World Health Organization. AIDS Epidemic Update December 2002. 12-1-2002. Report

3. McGhee JR, Czerkinsky C, and Mestecky J. Mucosal vaccines: An overview. In *Mucosal Immunology* (P.L.Ogra, J.Mestecky, M.E.Lamm, W.Strober, J.Bienenstock and J.R.McGhee, eds.), pp. 741-757, Academic Press, San Diego, 1999.

4. Lehner T, Wang Y, Cranage M, Bergmeier LA, Mitchell E, Tao L, Hall G, Dennis M, Cook N, Brookes R, Klavinskis L, Jones I, Doyle C, and Ward R. Protective mucosal immunity elicited by targeted iliac lymph node immunization with a subunit SIV envelope and core vaccine in macaques. *Nat.Med.* **2**: 767-775, 1996.

5. Lehner T, Bergmeier LA, Tao L, Panagiotidi C, Klavinskis LS, Hussain L, Ward RG, Meyers N, Adams SE, Gearing AJH, and Brookes R. Targeted lymph node immunization with simian immunodeficiency virus p27 antigen to elicit genital, rectal, and urinary immune responses in nonhuman primates. *J.Immunol.* **153**: 1858-1868, 1994.

6. Klavinskis LS, Bergmeier LA, Gao L, Mitchell E, Ward RG, Layton G, Brookes R, Meyers NJ, and Lehner T. Mucosal or targeted lymph node immunization of macaques with a particulate SIVp27 protein elicits virus-specific CTL in the genito-rectal mucosa and draining lymph nodes. *J.Immunol.* **157**: 2521-2527, 1996.

7. Marshall JL, Hoyer RJ, Toomey MA, Faraguna K, Chang P, Richmond E, Pedicano JE, Gehan E, Peck RA, Arlen P, Tsang KY, and Schlom J. Phase I study in advanced cancer patients of a diversified prime-and- boost vaccination protocol using recombinant vaccinia virus and recombinant nonreplicating avipox virus to elicit anti-carcinoembryonic antigen immune responses.

8. Jin X, Ramanathan M, Barsoum S, Deschenes GR, Ba L, Binley J, Schiller D, Bauer DE, Chen DC, Hurley A, Gebuhrer L, El Habib R, Caudrelier P, Klein M, Zhang LQ, Ho DD, and Markowitz M. Safety and immunogenicity of ALVAC vCP1452 and recombinant gp160 in newly human immunodeficiency virus type 1-infected patients treated with prolonged highly active antiretroviral therapy. *J.Virol.* **76**: 2206-2216, 2002.

9. Belshe RB, Stevens C, Gorse GJ, Buchbinder S, Weinhold K, Sheppard H, Stablein D, Self S, McNamara J, Frey S, Flores J, Excler JL, Klein M, Habib RE, Duliege AM, Harro C, Corey L, Keefer M, Mulligan M, Wright P, Celum C, Judson F, Mayer K, McKirnan D, Marmor M, and Woody G. Safety and immunogenicity of a canarypox-vectored human immunodeficiency virus Type 1 vaccine with or without gp120: a phase 2 study in higher- and lower-risk volunteers.

10. Gupta K, Hudgens M, Corey L, McElrath MJ, Weinhold K, Montefiori DC, Gorse GJ, Frey SE, Keefer MC, Evans TG, Dolin R, Schwartz DH, Harro C, Graham B, Spearman PW, Mulligan M, and Goepfert P. Safety and immunogenicity of a high-titered canarypox vaccine in combination with rgp120 in a diverse population of HIV-1-uninfected adults: AIDS Vaccine Evaluation Group Protocol 022A.

11. AIDS Vaccine Evaluation Group 022 Protocol Team. Cellular and Humoral Immune Responses to a Canarypox Vaccine Containing Human Immunodeficiency Virus Type 1 Env, Gag, and Pro in Combination with RGP120. *J.Infect.Dis.* **183**: 563-570, 2001.

12. Ferrari G, Humphrey W, McElrath MJ, Excler JL, Duliege AM, Clements ML, Corey LC, Bolognesi DP, and Weinhold KJ. Clade B-based HIV-1 vaccines elicit cross-clade cytotoxic T lymphocyte reactivities in uninfected volunteers. *Proc.Natl.Acad.Sci.U.S.A* **94**: 1396-1401, 1997.
